# Thiocoumarin‐based Au(I) Complexes and Au(0) Systems over TiO_2_ as Hybrid Photocatalysts for Hydrogen Generation under UV–Vis Light

**DOI:** 10.1002/advs.202404969

**Published:** 2024-11-05

**Authors:** Asier Agrelo‐Lestón, Jordi Llorca, Elizabeth Martínez, Inmaculada Angurell, Laura Rodríguez, Lluís Soler

**Affiliations:** ^1^ Center for Research in Multiscale Science and Engineering and Department of Chemical Engineering Institute of Energy Technologies Universitat Politècnica de Catalunya (UPC) EEBE Eduard Maristany 10–14 Barcelona 08019 Spain; ^2^ Departament de Química Inorgànica i Orgànica Secció Química Inorgànica Universitat de Barcelona Martí i Franquès 1 Barcelona 08028 Spain; ^3^ Institut de Nanociència i Nanotecnologia (IN2UB) Universitat de Barcelona Avda Diagonal 647 Barcelona 08028 Spain

**Keywords:** gold complexes, gold nanoparticles, heterogeneous photocatalysis, thiocoumarin, titanium dioxide

## Abstract

This work focuses on the photocatalytic production of hydrogen from the photodehydrogenation of ethanol using several gold(I) complexes and gold(0) systems over titanium dioxide (P90 TiO_2_) as hybrid photocatalysts. The photocatalytic systems are composed of at least one coumarin‐based ligand, which can enhance the photocatalytic activity by its photon‐absorbing capacity due to its chromophore properties. The photocatalytic behavior for hydrogen generation of the studied samples is compared under UV–vis light setting the total gold‐based co‐catalyst loading at 1 wt% onto the TiO_2_ photocatalysts and when the gold content is maintained at 0.25 wt%. The incorporation of gold co‐catalysts results in an enhancement of hydrogen production up to 2.7 times compared to a conventional Au/TiO_2_ reference sample. The results show an increase in the total hydrogen production under UV–vis light due to the combined presence of coumarin chromophore, gold‐based co‐catalysts, and gold plasmonic nanoparticles. A deep characterization of the samples from each group is performed by UV–vis spectroscopy, XPS, HRTEM, and HAADF‐STEM, observing the presence of plasmonic gold nanoparticles for sample “AuL1NPs” and the reduction of the gold present in sample “AuL1a,” which explains the highest observed hydrogen production rates of this study.

## Graphical abstract

This work explores hydrogen production through ethanol photodehydrogenation using coumarin‐based gold(I) complexes and gold(0) systems over titanium dioxide. The addition of coumarin‐based ligands to form these gold co‐catalysts is significantly, ca. three times, enhanced hydrogen production compared to conventional Au/TiO_2_ photocatalyst. Characterization techniques reveal the presence of plasmonic gold nanoparticles which have broaden the light absorption capacity to visible light.

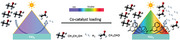

## 1 Introduction

Hydrogen (H_2_) is considered a highly promising energy carrier for the decarbonization of the global energy system.^[^ ^1^ ^]^ It is currently projected to be a leader in the energy sector in both stationary and mobile applications.^[^ ^2^ ^]^ Nevertheless, the use of H_2_ for the decarbonization of the energy system will not proceed if it is not based on a sustainable H_2_ production system.^[^ ^3^ ^]^ Nowadays, most of the produced H_2_ for industrial applications comes from the reforming of natural gas,^[^ ^4^, ^5^, ^6^, ^7^, ^8^ ^]^ with its associated CO_2_ emissions.

Photocatalytic H_2_ production is currently attracting a lot of interest due to its potential for the use and storage of solar energy, which in the future could make a major contribution to mitigate high energy demands and reduce greenhouse gas emissions.^[^ ^9^ ^]^ Despite significant research in the field of photocatalysis, there is still a significant gap between lab‐scale working systems and their industrial applications.^[^ ^9^ ^]^ One of the main challenges of the photocatalytic processes is to find a material able to exploit a large part of the solar spectrum, as well as other characteristics such as high photoreaction efficiency, stability, and low cost.^[^ ^10^, ^11^ ^]^ There are different approaches to improve the catalytic activity of a photocatalyst, where the use of co‐catalysts stands out as an effective way to reduce the recombination rate of the electron‐hole pairs.^[^ ^11^ ^]^ On several occasions, the integration of metallic complexes to a photocatalysts in order to enhance its photoactivity has been studied.^[^ ^12^, ^13^ ^]^ In this kind of hybrid systems the particular interaction between the catalysts and the photosensitizer, in combination with the nature of the metal, plays a key role in its resultant performance and stability.^[^ ^13^ ^]^ These systems are, typically, based on transition metals such as copper, nickel, cobalt, zinc, iridium, or ruthenium.^[^ ^14^, ^15^, ^16^ ^]^ The introduction of these co‐catalysts allows the absorption of visible light and the possibility of transferring excited stated to TiO_2_, thus, enhancing its photocatalytic activity under solar radiation.^[^ ^17^, ^18^ ^]^ This strategy not only broadens the utilization of solar energy but also offers the potential for controlling the photocatalytic properties by the design of the metal‐ligand environment.^[^ ^19^ ^]^ For instance, Kong et al.^[^ ^20^ ^]^ reported different Co, Ni, and Cu‐based metal complex loaded over TiO_2_ photocatalysts with high stability and improved activity. Xao et al.^[^ ^21^ ^]^ developed some aldehyde functionalized Iridium (III) complexes to photosensitize titania for hydrogen production using blue and green light. Despite the advancements in the last 10 years, the most of photocatalytic technologies for H_2_ production are still at low technology readiness levels (TRLs) TRL 1–3.^[^ ^22^, ^23^ ^]^

Here, we report a research study to follow up the work by Aguiló et al.,^[^ ^3^ ^]^ in which some gold(I) complexes containing a coumarin and a phosphane (DAPTA, 3,7‐diacetyl‐ 1,3,7‐triaza‐5‐phosphabicyclo[3.3.1]nonane or PPh_3_, triphenylphosphine) ligands were prepared and tested for the photogeneration of H_2_ under UV light, showing better performances than a conventional Au/TiO_2_ prepared by impregnation. In the present work, the tested complexes are based on two different coumarin ligands, which could have an influence on the photoinduced electron transfer of gold to titania due to its chromophoric properties, as they could act as photon capturers broadening effectively the absorption capacity of the photocatalyst to the visible region. Both coumarin ligands contain thiol groups at the terminal positions to facilitate the coordination with gold. The main difference among them is the proximity of the coumarin to the metal center, which was also evaluated in this work. The effect of the DAPTA ligand on hydrogen production was also studied since the presence of the phosphane in Au(I) complexes could enhance the charge transfer from the semiconductor bands to the Au active site.^[^ ^3^, ^24^ ^]^ The same thio‐coumarin ligands have been used to stabilize Au(0) nanoparticles that were also studied for comparison purposes. A comparison between co‐catalyst with preformed stabilized small‐size nanoparticles and single atom catalyst was also carried out. All the hybrid photocatalysts have been tested in the generation of hydrogen at room temperature under dynamic conditions in gas phase and irradiated with UV–vis light, with the aim of using the visible spectrum for efficient production of H_2_.

## 2 Results and Discussion

### 2.1 Characterization of the Synthesized Complexes and Systems

**Figure 1:**
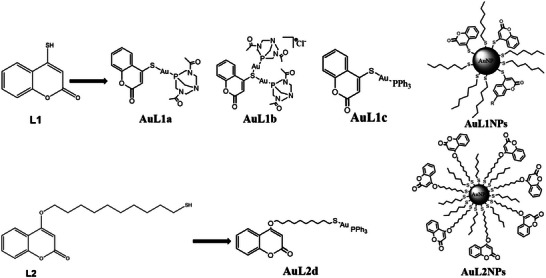
Molecular structure of L1 and L2 and the corresponding Au(I)‐complexes and Au(0)‐systems.

Two different thiocoumarin‐based ligands (4‐mercapto‐2H‐chromen‐2‐one and 4‐((10‐mercaptodecyl)oxy)‐2H‐chromen‐2‐one) were synthesized and named L1 and L2, respectively. Both L1 and L2 ligands contain a thiol group to facilitate the coordination with the gold. The main difference among L1 and L2 thiocoumarins is the incorporation of a ten‐carbon chain in ligand L2 to increase the distance from the coumarin group to the gold site, as stated in **Figure** **1**. Six different Au‐based co‐cocatalysts were synthesized and studied, which are included in Figure 1. Two main groups can be distinguished within the six studied co‐catalysts: those based on Au(I) (named AuL1a, AuL1b, AuL1c, and AuL2d; see their chemical formulas in Figure 1) and those based on preformed gold nanoparticles (named AuL1NPs and AuL2NPs; see their chemical formulas in Figure 1). The first studied co‐catalyst, named AuL1a, is a single‐atom co‐catalyst composed of a gold atom coordinated to a thiocoumarin (L1 ligand) and a DAPTA ligand, in a thiocoumarin:Au‐DAPTA 1:1 molar ratio. The second studied co‐catalyst, named AuL1b, is a co‐catalyst similar to AuL1a, but AuL1b included a thiocoumarin:Au‐DAPTA 1:2 molar ratio, i.e., it is composed of two gold atoms, each Au atom was coordinated to a DAPTA ligand and both Au atoms were bonded to the same sulfur atom of the thiocoumarin (L1 ligand). The third studied co‐catalyst, named AuL1c, is similar to AuL1a but it has a triphenylphosphine (PPh_3_) ligand instead of the DAPTA ligand present in AuL1a. The fourth studied co‐catalyst, named AuL2d, incorporated a ten‐carbon chain in the thiocoumarin ligand (labeled L2 ligand), which increased the number of carbon atoms between the thiocoumarin group and the gold center. AuL2d also included a PPh_3_ as a ligand of the Au atom. The fifth studied co‐catalyst, named AuL1NPs, was a gold nanoparticle‐based system, which was stabilized by three thiocoumarin (L1 ligands) directly coordinated to the gold site and five hydrocarbon chains (six carbon atoms per each hydrocarbon chain) bonded to the Au nanoparticle through a sulfur atom per chain. The sixth studied co‐catalyst, named AuL2NPs, included seven L2 ligands coordinated with the Au nanoparticle and eight hydrocarbon chains (six carbon atoms per each hydrocarbon chain) bonded to the Au nanoparticle through a sulfur atom per chain.

To follow and confirm the successful obtention of the thiocoumarin‐based ligands we performed ^1^H NMR, IR spectroscopy and MS spectrometry. For L1, by ^1^H NMR the thiol group could be observed as a singlet at 3.86 ppm (Figure S1, Supporting Information). The same thiol group was seen as a middle intensity band at 2477 cm^−1^ in IR spectrum due to the SH stretching (Figure S2, Supporting Information). In the case of L2, the obtention of the ligand could be easily followed by ^1^H NMR due to the signal of the methylene group closer to the ‐SR shifts from 2.86 to 2.53 ppm, and changes in its multiplicity from a triplet to a pseudo quartet with a coupling constant ≈8 Hz as well as the ‐SH group is obtained (Figure S3, Supporting Information). In the IR spectrum, it could be observed a weak band at 2575 cm^−1^ corresponding to the SH stretching (Figure S4, Supporting Information), and ESI(+) mass spectrometry also confirmed the obtention of the ligand (m/z: 335.17 [M+H]^+^, Figure S5, Supporting Information).

All synthesized L1 and L2 containing Au(I) complexes (AuL1a, AuL1b, AuL1c, and AuL2d, see Scheme 2 in section Synthesis of Au(0)‐based systems) were obtained with moderate yields and characterized by IR, NMR, and MS Spectrometry. The absence of the SH stretching in the IR spectra (Figures S6–S9, Supporting Information) suggested the coordination of the thiolate group in all L1 containing Au(I) complexes, which was lately confirmed by ^1^H NMR as the disappearance of the terminal thiol proton was observed at 3.86 ppm as a singlet (Figures S10–S12, Supporting Information, for L1 ligand) upon the coordination of the metal. This result was a clear indication of the successful formation of the complexes.

The ^1^H NMR signals corresponding to the coumarin shifted significantly respect to free L1, especially those protons closer to the thiol group, which is a clear indication of the successful coordination to the metal center. H5 shifted from 7.64 ppm to 8.26 for AuL1c and to 8.14 for AuL1a and AuL1b. H3 shifted from 6.42 to 7.01 ppm and 6.86 ppm for PPh_3_‐containing (AuL1c) and DAPTA‐containing complexes (AuL1a and AuL1b), respectively. DAPTA signals were observed in the range of 5.80–3.67 ppm for complexes AuL1a and AuL1b. The integration of the signals confirmed the DAPTA stoichiometry of the complexes, 1:1 and 1:2 for AuL1a and AuL1b (Figures S10 and S11, Supporting Information).

The assignments of the ^13^C{^1^H} NMR signals were made with ^1^H‐^13^C heteronuclear single quantum coherence spectroscopy (HSQC) experiments (Figures S13–S17, Supporting Information). C5 and C3 signals of the coumarin were downfield shifted relative to the starting L1 ligand, while C6‐8 were upfield shifted. The ^31^P{^1^H} NMR spectra (Figures S18–S20, Supporting Information) present a unique resonance for all complexes, at 38.3 ppm, −24.8, and −28.4 ppm, for AuL1c, AuL1a, and AuL1b respectively. It is worth noting that the signal of AuL1c is up‐field shifted (4 ppm) compared with AuL1a. A single signal of ^31^P in the NMR spectrum provided a clear sign of the obtention of a pure product since it demonstrated a single environment around P. The mass spectra (ESI(+)) presented for all the neutral compounds the molecular peak [M+H]^+^, and for the cationic complex the peak [M‐Cl]^+^ (Figures S21–S23, Supporting Information), again as evidence of the successful synthesis of the complexes.

AuL2d synthesis was confirmed by IR,^1^H NMR, and ^31^P{^1^H} NMR, and MS spectrometry (Figures S24–S26, Supporting Information). The coordination of L2 to the gold atom was associated with the shifting downfield of the signal of the CH_2_S (from 2.53 to 3.01 ppm), and again to the change in the multiplicity of the signal. ^31^P{^1^H} NMR showed a broad signal at 36.2 ppm, and ESI(+) MS spectra a peak at 793.22 attributed at [M+H]^+^.

Au(0) based systems where characterized by ^1^H NMR, IR spectroscopy, TGA, and TEM. The ^1^H NMR of systems AuL1NPs and AuL2NPs revealed the presence of L1 and L2, respectively. As expected, the signals of the protons closer to the surface of the gold nanoparticles were wide and no clear (Figures S27 and S28, Supporting Information). Therefore, to quantify the proportion of L1 or L2 to the 1‐hexanethiol, which stabilizes gold nanoparticles, a decomplexation reaction using iodine in methanol was performed.^[^ ^25^ ^] 1^H NMR measurements were performed to the disulphide ligands (Figures S27 and S28, Supporting Information), which showed a ratio of 1‐hexanethiol to ligand L1 of 1:0.07 and a ratio of 1‐hexanethiol to ligand L2 of 1:0.8 in the systems AuL1NPs and AuL2NPs, respectively, confirming the successful synthesis of both Au(0) based systems.

The IR spectra of the nanoparticles (Figures S29 and S30, Supporting Information) also revealed the presence of the coumarin moieties through the existence of bands of the C═O stretching at 1676 and 1645 cm^−1^ in the case of AuL1NPs, and at 1717 cm^−1^ in the case of AuL2NPs. It is worth noting that an important shift of the C═O band was observed in AuL1NPs, indicating that the thiol group is binding gold atoms in agreement with the values obtained in gold(I) complexes described before. In the case of AuL2NPs, no significative change in the C═O vibrational frequency was observed, related to the longer distance between the coumarin moiety and the metal center.

Thermogravimetric analysis of both Au(0) based showed gold contents of 86% and 72% for AuL1NPs and AuL2NPs, respectively (Figure S31, Supporting Information). The nanoparticle size measurements made from TEM images obtained for both Au(0) systems (Figure S32, Supporting Information) revealed a mean size of 3.1 ± 0.8 nm.

### 2.2 Photocatalytic Activity

**Figure 2:**
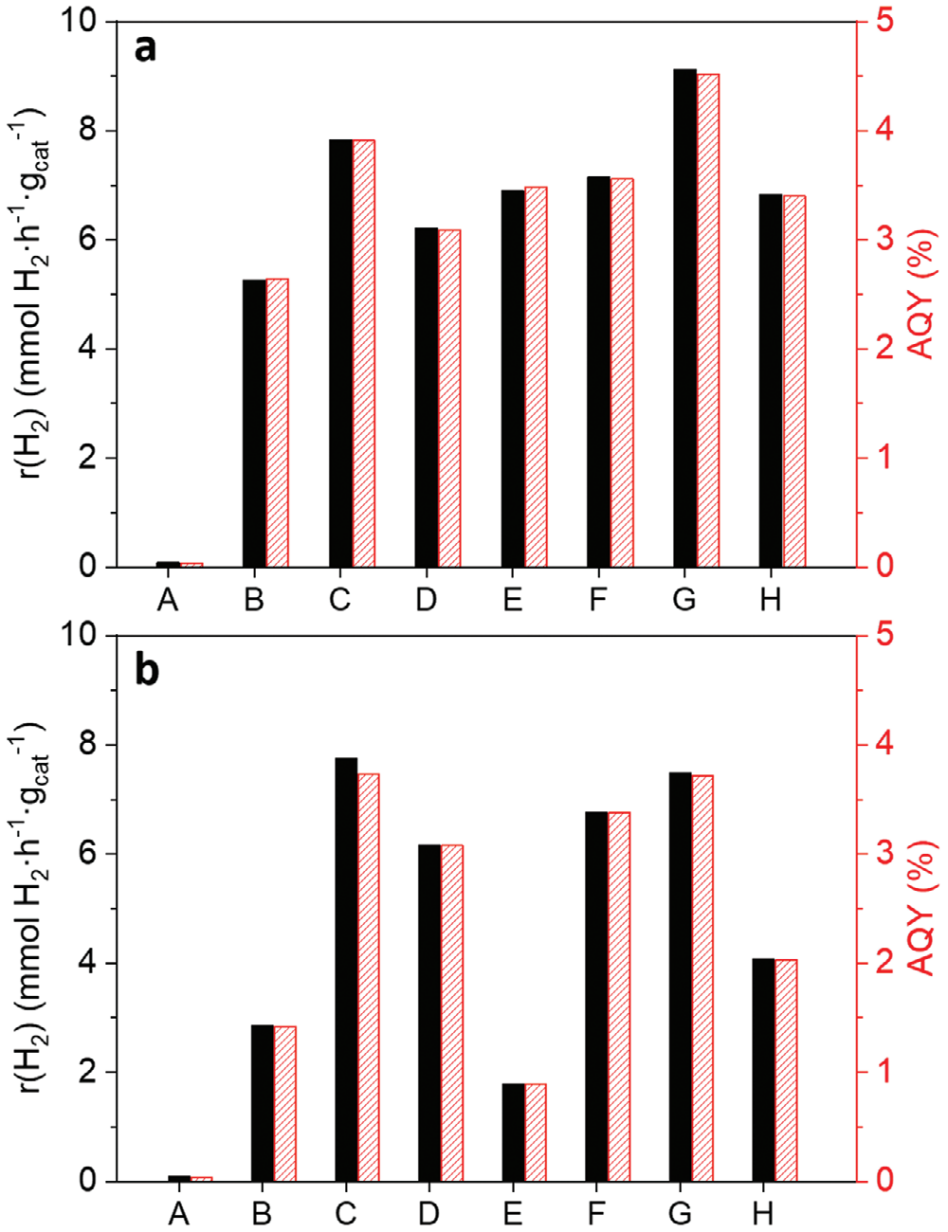
Hydrogen photoproduction rates (black bars) and AQY (red bars) of the different samples under (a) UV light irradiation with Au(I)‐complex or Au(0)‐system loading of 1 wt% over TiO_2_ P90: (A) TiO_2_, (B) Au‐1/TiO_2_, (C) AuL1a/TiO_2_ (Au 0.32 wt%), (D) AuL1b/TiO_2_ (Au 0.37 wt%), (E) AuL1c/TiO_2_ (Au 0.31 wt%), (F) AuL2d/TiO_2_ (Au 0.25 wt%), (G) AuL1NPs/TiO_2_ (Au 0.86 wt%), (H) AuL2NPs/TiO_2_ (Au 0.72 wt%). (b) under UV light irradiation with gold loading of 0.25 wt%: (A) TiO_2_, (B) Au‐0.25/TiO_2_, (C) AuL1a/TiO_2_, (D) AuL1b/TiO_2_, (E) AuL1c/TiO_2_, (F) AuL2d/TiO_2_, (G) AuL1NPs/TiO_2_, (H) AuL2NPs/TiO_2_.

In this work, hydrogen molecules were produced through the well‐known photocatalytic dehydrogenation of ethanol (C_2_H_5_OH → CH_3_CHO + H_2_),^[^ ^26^ ^]^ where acetaldehyde was also obtained as a byproduct of the reaction in a H_2_:acetaldehyde stoichiometric ratio of 1:1. To assess the photocatalytic behavior of the synthesized Au(I)‐complexes and Au(0)‐systems, which were employed as co‐catalysts in the present work, we first tested all the hybrid Au(I)‐complex‐TiO_2_ and Au(0)‐systems‐TiO_2_ for hydrogen photogeneration, fixing a total co‐catalyst load of 1 wt% over TiO_2_ P90 in each case, and compared them to the Au‐1/TiO_2_ reference sample with a gold loading of 1 wt% and also to bare TiO_2_‐P90, which actually showed a very low photocatalytic activity (0.09 mmol h^−1^ g_cat_^−1^) due to the well‐known fast recombination of the photogenerated electron‐hole pairs in pristine TiO_2_.^[^ ^27^ ^]^ As depicted in **Figure** **2**a, all the prepared hybrid photocatalysts resulted to be more active in hydrogen production compared to pristine TiO_2_‐P90 and the reference sample Au‐1/TiO_2_. Specifically, AuL1a/TiO_2_ and AuL1NPs/TiO_2_ resulted to be the most active samples under irradiation of UV light (see (C) and (G) results in Figure 2a, respectively), reaching hydrogen production rates 1.5 and 1.7 times greater than a conventional Au‐1/TiO_2_ reference sample (see (B) in Figure 2a). Nevertheless, the variation of the molecular weights of the ligands among the different synthesized Au(I)‐complexes and Au(0)‐systems caused a difference in the gold content when the same amount of co‐catalyst was impregnated over TiO_2_ (see differences of wt% Au contents in Figure 2a). For this reason, we prepared and tested a second set of hybrid photocatalytic samples with an equal gold mass loading of 0.25 wt%, in order to compare the different samples under the same gold basis and to explore the influence of the different ligands on the photocatalytic activity. As shown in Figure 2b, the tendency toward hydrogen production of the hybrid photocatalytic samples with Au 0.25 wt% remained similar to the previous set of samples containing 1 wt% of Au(I)‐complexes or Au(0)‐systems, with the exception of the sample impregnated with AuL1c (see (E) in Figure 2b), which suffered a large decrease in the observed hydrogen production rate when the Au content was fixed to 0.25 wt%. Under the same gold basis, AuL1a/TiO_2_ and AuL1NPs/TiO_2_ kept as the most photoactive samples under UV light irradiation (see (C) and (G) results in Figure 2b, respectively), reaching hydrogen production rates 2.7 and 2.6 times greater than a conventional Au‐0.25/TiO_2_ reference sample.

**Figure 3:**
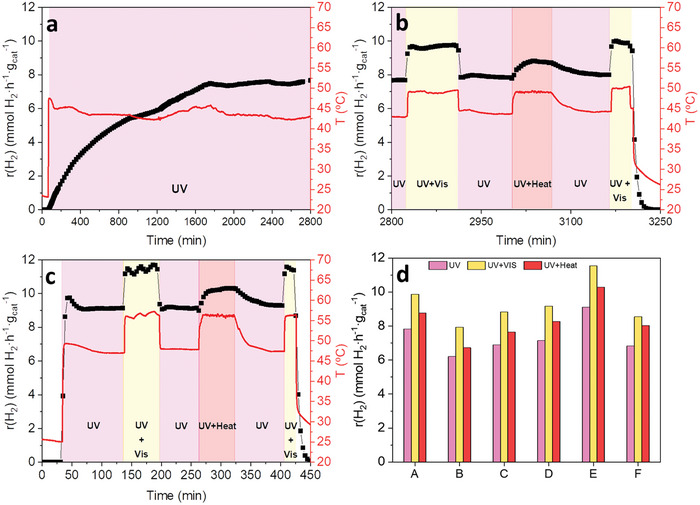
Hydrogen generation rates under different light irradiation conditions with Au(I)‐complex or Au(0)‐system loading of 1 wt% over TiO_2_ P90. (a) AuL1a/TiO_2_ (Au 0.32 wt%) activation phase. (b) AuL1a/TiO_2_ (Au 0.32 wt%) analysis of light and heat effect. (c) AuL1NPs/TiO_2_ (Au 0.86 wt%). (d) Hydrogen generation rates at different light irradiation combinations, UV (purple), UV + vis (yellow), UV + heat (red): (A) AuL1a/TiO_2_ (Au 0.32 wt%), (B) AuL1b/TiO_2_ (Au 0.37 wt%), (C) AuL1c/TiO_2_ (Au 0.31 wt%), (D) AuL2d/TiO_2_ (Au 0.25 wt%), (E) AuL1NPs/TiO_2_ (Au 0.86 wt%), (F) AuL2NPs/TiO_2_ (Au 0.72 wt%).

To gain more insight into the influence of the chemical structure of the studied ligands of the Au(I)‐complexes on the photocatalytic activity, we also compared the observed photocatalytic activities of the different tested hybrid Au(I)‐complexes/TiO_2,_ i.e., AuL1a, AuL1b, AuL1c, and AuL2d containing the same Au basis (0.25 wt%). Although all the tested Au‐complexes included a coumarin ligand in each complex, the tested complexes differed in the distance (i.e., number of carbon atoms in the chain) between the coumarin group and the gold site (L1 or L2) and they also differed in both the number and/or type of the other ligands (DAPTA, PPh_3_) different than the coumarin. Therefore, a careful discussion of the results is given below. The effect of the presence of DAPTA ligand on the photocatalytic activity can be observed comparing the hydrogen production results obtained with samples AuL1a/TiO_2_ (containing the DAPTA phosphane ligand, see sample (C) in Figure 2b) versus AuL1c/TiO_2_ (containing the PPh_3_ phosphane, see sample (E) in Figure 2b). The presence of PPh_3_ had a negative effect on its hydrogen photoproduction performance, as observed comparing (C) versus (E) results in Figure 2b. Additionally, the substitution of the DAPTA group by a PPh_3_ led to shorter induction periods to reach a steady state on hydrogen production (47 h for AuL1a/TiO_2_ against 15 h for AuL1c/TiO_2_, respectively), as shown in **Figures** **3**a and S33a (Supporting Information), which could be related to a more significant reduction of the gold atoms in AuL1a/TiO_2_, when DAPTA ligand was present in the complex.

The stoichiometric ratio of the coumarin:Au‐DAPTA in the tested Au‐complexes could have an impact on hydrogen photoproduction. Consequently, the activity of AuL1a/TiO_2_ and AuL1b/TiO_2_ hybrid photocatalysts was compared (see (C) and (D) results in Figure 2b). These two complexes differ in the amount of Au‐DAPTA bonded to the thiocoumarin, since AuL1a had a stoichiometry of 1:1 coumarin:Au‐DAPTA whereas AuL1b had a stoichiometry of 1:2 coumarin:Au‐DAPTA. As stated in Figure 2, the photocatalytic performance of AuL1a/TiO_2_ was higher than AuL1b/TiO_2_ in both basis for comparison (1 wt% total amount of complex, Figure 2a, and 0.25 wt% total amount of Au, Figure 2b). Therefore, a stoichiometric ratio of 1:1 coumarin:Au‐DAPTA site benefited the photocatalytic activity of the hybrid samples.

Moreover, the number of carbon atoms between the coumarin and the gold site could also have influence on the photocatalytic activity. Hence, we compared the obtained results for AuL1c/TiO_2_ and AuL2d/TiO_2_ samples (see (E) and (F) in Figure 2b), because AuL2d/TiO_2_ incorporated a long carbon chain to separate the coumarin group and the Au site. As shown in Figure 2b, the hydrogen photoproduction was higher employing AuL2d/TiO_2_ than AuL1c/TiO_2_ (see (F) and (E) in Figure 2b, respectively). Therefore, a higher number of carbon atoms between the coumarin and the gold site enhanced the photocatalytic activity. Additionally, according to the above‐mentioned comparison between AuL1a/TiO_2_ and AuL1c/TiO_2_ (see (C) vs (E) results in Figure 2b), the negative effect on the hydrogen photoproduction due to the presence of PPh_3_ disappeared with the addition of a long hydrocarbon chain between the coumarin and the gold site. A possible hypothesis could be that the PPh_3_ group was producing a shadowing effect, in which the coumarin was not efficiently acting as a photon capturer, because the presence of a close PPh_3_ group could either impede an appropriate light interaction of the coumarin group and/or the combined interaction of the coumarin group, the Au site and the TiO_2_ particle under light irradiation. So, an increase of the number of carbon atoms between the coumarin and the PPh_3_ group prevented the shadowing effect and benefited the photocatalytic activity. Actually, a comparison of AuL1a vs AuL2d (see (C) vs (F) in Figure 2b) demonstrated that the replacement of a DAPTA ligand by a PPh_3_ ligand when the coumarin is separated from the Au site by a higher number of carbon atoms (L2 ligand instead of L1 ligand) resulted in similar photocatalytic hydrogen production rates. In fact, the photocatalytic activity was slightly higher for the AuL1a (containing a DAPTA ligand) than the AuL2d (containing a “coumarin‐separated” PPh_3_ ligand), which could point out to a particular combined effect of the “coumarin + Au site + DAPTA + TiO_2_” under light irradiation. Nevertheless, the combined effect of the “coumarin + ten carbon chain + Au site + PPh_3_” shows a comparable photocatalytic activity to AuL1a, concluding that the DAPTA ligand would play a less important role in the hybrid photocatalyst behavior. On the other hand, the observed positive effect of a longer hydrocarbon chain between Au and the coumarin group changed when we used hybrid photocatalysts based on gold nanoparticles (AuL1NPs/TiO_2_ and AuL2NPs/TiO_2_) instead hybrid photocatalysts with Au‐single atom complexes. In the case of Au nanoparticle‐based systems, a longer hydrocarbon chain between the coumarin group and the Au nanoparticle provoked a negative effect on the photocatalytic hydrogen production, as shown in Figure 2b for (G) and (H) results, which correspond to AuL1NPs/TiO_2_ and AuL2NPs/TiO_2_, respectively.

As described in the experimental section, the photocatalytic tests were divided into different steps to evaluate the changes on hydrogen production provoked by different light irradiation conditions (see Scheme S1, Supporting Information). Figure 3 shows the hydrogen production rate profiles of the most active samples, AuL1a/TiO_2_ and AuL1NPs/TiO_2_, based on Au(I) and Au(0), respectively. Actually, all Au(I)‐based hybrid samples showed a long induction period (15–47 h) in which there was a continuous increase of the hydrogen production rate until reaching a steady state (Figure 3a; Figures S33–S35, Supporting Information). As further discussed in section 2.3, this effect could be attributed to a progressive reduction of the Au(I) species to form Au(0) nanoparticles. In the case of samples containing Au(0) nanoparticles, there was not observed any induction period during the photocatalytic experiment under UV‐light irradiation(Figure 3c; Figure S36, Supporting Information).

All the explored hybrid samples in this work showed photocatalytic response to visible light irradiation when UV light was also present (third step), being samples AuL1a/TiO_2_ and AuL1NPs/TiO_2_ the most active ones (Figure 3d). These samples reached H_2_ production rates of ≈9.9 and 11.5 mmol h^−1^ g_cat_^−1^, respectively, which means a relative improvement of 25% in both cases, compared to the H_2_ production rates observed under UV irradiation. An increase of the reaction temperature was observed when both UV and visible light sources were employed simultaneously. This temperature increase was attributed to the intrinsic thermal irradiation of the visible light source employed in the experiments. Consequently, an extra control experiment was performed applying external heat to the reaction system when it was illuminated with UV light (Scheme S1, Supporting Information, fifth step), in order to assess the contribution of the increase of temperature on the hydrogen production rate, with the absence of visible light irradiation. The obtained H_2_ production rate values when applying UV light together with an external heat source, reaching the same value of observed temperature when the sample was irradiated with both UV+ visible light sources, were lower than the H_2_ production rate obtained under both UV and visible light irradiation. Therefore, the temperature increase was excluded as the main reason for the improvement of the observed photocatalytic catalytic activity and it was confirmed that all the hybrid catalysts based on TiO_2_ with Au(I) complexes and Au(0) systems tested in the present study showed a higher activity on the hydrogen photoproduction rates under both ultraviolet and visible light irradiation than under only ultraviolet light irradiation (see Figure 3d).

### 2.3 Characterization of the Hybrid Photocatalysts

We carried out a detailed characterization before and after the photoreaction of the two most active samples for the photogeneration of hydrogen, AuL1a/TiO_2_ and AuL1NPs/TiO_2_, which were based on coumarin‐Au(I)‐DAPTA complexes and coumarin‐Au(0)‐nanoparticles, respectively, in order to shed light on the reasons for their particular catalytic behavior.

**Figure 4:**
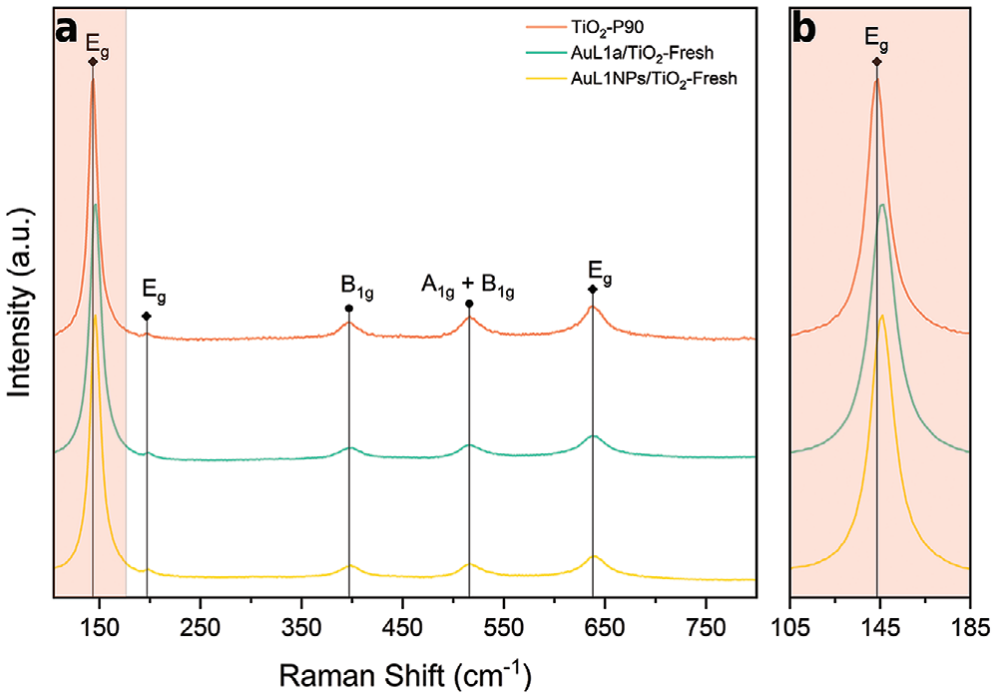
Raman spectra of TiO_2_‐P90, AuL1a/TiO_2_ and AuL1NPs/TiO_2_. (a) Complete Raman spectra. (b) zoom of the highlighted area.

**Figure** **4** shows the Raman spectra of the fresh samples (before reaction) compared to pure TiO_2_ P90. The five typical peaks related to anatase phase^[^ ^28^ ^]^ are observed in all samples at 143.6 (E_g_), 196.4 (E_g_), 396.7 (B_1_ _g_), 516.2 (A_1_ _g_ + B_1_ _g_), and 638.3 cm^−1^ (E_g_). Rutile‐related peaks are not seen in any of the samples. A small shift to higher wavenumbers can be observed in both impregnated samples compared to pristine TiO_2_ P90 (143.6 vs 145.8 cm^−1^). This shift is related to crystalline defects formed at the contact area of gold and TiO_2_, which can act as charge carrier trap sites.^[^ ^29^, ^30^, ^31^ ^]^

**Figure 5:**
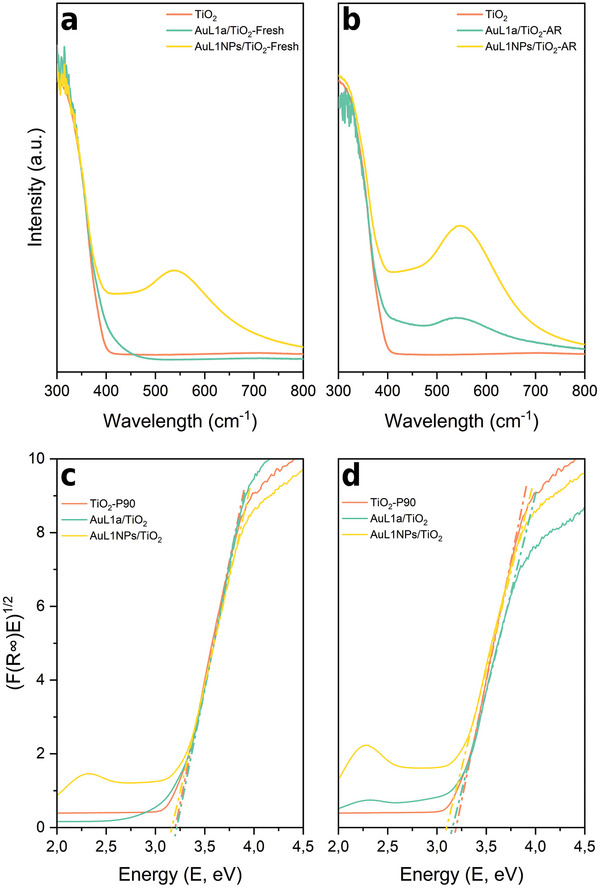
UV–vis spectra for the (a) fresh samples and (b) After reaction samples. Tauc plots of. (c) Fresh samples. (d) After reaction samples.

UV–vis spectroscopy was used to determine the possible plasmonic bands of gold co‐catalysts loaded over TiO_2_ and to determine the band gap of the studied photocatalysts by means of Tauc plots. **Figure** **5**a,b shows UV–vis spectra of both samples before and after the reaction. There is significant change for AuL1a/TiO_2_ sample in the after‐reaction spectra, as a surface plasmon resonance band of the gold appeared ≈535 nm, which confirmed the previously mentioned reduction of the gold present in this complex to form Au(0) nanoparticles. Accordingly, this sample suffered a macroscopic color changes of the powder before and after the photocatalytic reaction from white to brownish color (Figure S37a, Supporting Information) as a consequence of the Au reduction. In the case of AuL1NPs/TiO_2_, the surface plasmon resonance band of gold is present both before and after the reaction, as this system was based on preformed gold nanoparticles (Figure 5a,b). Nevertheless, there is a small shift from 540 nm (before reaction) to 547 nm (after reaction). It is well known that the localized surface plasmon resonance effect of gold nanoparticles is size‐ and shape‐dependent, so this minor shift could be attributed to a slight agglomeration of gold nanoparticles under photoreaction.^[^ ^32^ ^]^

The bandgap energy values calculated from Tauc plots (Figure 5c,d) on the fresh samples were 3.20, 3.21, and 3.17 eV for pure TiO_2_ P90, AuL1a/TiO_2_, and AuL1NPs/TiO_2_, respectively. All three values remain quite similar, being AuL1NPs/TiO_2_ slightly lower than bare TiO_2_. After reaction, the obtained values were 3.16 eV for AuL1a/TiO_2_ and 3.10 eV for AuL1NPs/TiO_2_, which are lower bandgap values compared to bare TiO_2_. A lower band gap value is indicative of the surface plasmon resonance effect of the metal present on the anchored co‐catalysts, which enhances light absorption on visible spectra.^[^ ^33^, ^34^, ^35^ ^]^

**Figure 6:**
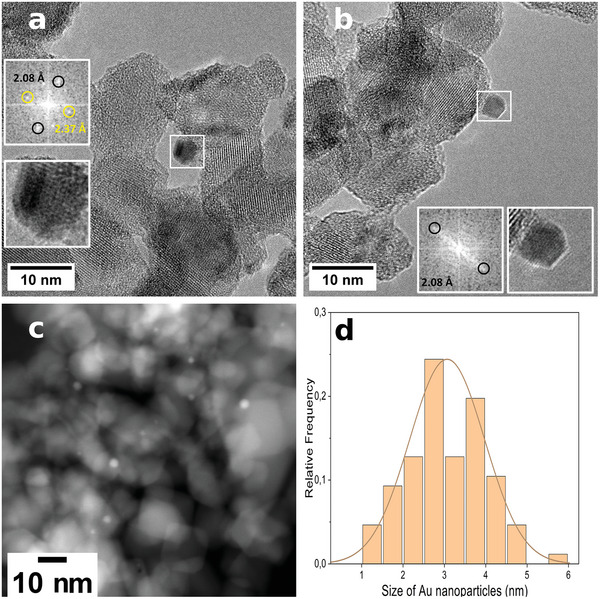
HRTEM (a,b), HAADF‐STEM, (c) and nanoparticle size distribution (d) of AuL1a/TiO_2_ after reaction.

The sample AuL1a/TiO_2_ was examined after reaction by HRTEM (**Figure** **6**a,b) and HAADF‐STEM (Figure 6c). HAADF‐STEM images clearly confirmed the formation of gold nanoparticles (bright particles) after reaction with a mean size of 3.1 ± 0.9 nm (Figure 6d). HRTEM images also allowed to unambiguously identify the presence of Au metal nanoparticles. Lattice fringes at 2.08 and 2.37 Å corresponding to the (200) and (111) crystallographic planes of Au, respectively, were measured in the nanoparticles with high electron contrast (see insets in Figure 6a,b). From the HRTEM analysis we could also confirm a strong contact between Au nanoparticles and TiO_2_. Since this sample was based on Au(I), we deduce that the induction period observed on the photocatalytic experiments (see Figure 3a; Figures S33–S35, Supporting Information) could be a consequence of the reduction of those gold atoms while their coordination environment changed. Possible changes in the co‐catalyst coordination environment were consistent with the obtained C/Ti ratios from XPS spectra in Table S1 (Supporting Information), which will be further discussed. Regarding the tested fresh hybrid photocatalyst samples, we expected the co‐catalyst to be coordinated to the TiO_2_, probably through the oxygen atom from the carbonyl group of the thiocoumarin, as noticed in previous work by Aguiló et al.^[^ ^3^ ^]^

**Figure 7:**
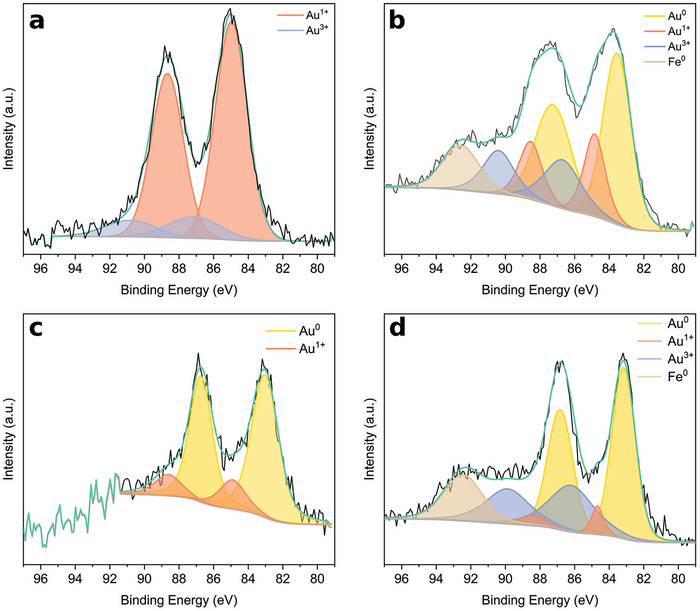
XPS spectra of fresh and after reaction samples. (a) AuL1a/TiO_2_‐fresh, (b) AuL1a/TiO_2_‐after reaction, (c) AuL1NPs/TiO_2_‐fresh, (d) AuL1NPs/TiO_2_‐after reaction.

X‐ray Photoelectron Spectroscopy (XPS) was used to analyze the oxidation state of gold in both samples. In this particular characterization, the gold content of the samples was increased to 1.5 wt% to ensure metallic concentration values above the detection limit of the XPS instrument. **Figure** **7**a,b corresponds to the Au 4f region of sample AuL1a/TiO_2_ before and after the reaction. As expected, before the photocatalytic reaction there was no presence of metallic gold in the complex based on Au(I). The gold 4f peaks shape suggested the presence of gold in two oxidation states. The peak located at 84.9 eV confirmed the existence of Au(I),^[^ ^36^, ^37^ ^]^ while peak at 87.1 eV refers to the presence of Au(III).^[^ ^38^, ^39^, ^40^ ^]^ In the post‐reaction sample a reduction of the Au(I) peak was observed together with the appearance of a peak at 83.6 eV corresponding to metallic gold, which confirmed the reduction of the gold contained in the AuL1a/TiO_2_ sample as discussed above. Together with these peaks there is also a 4f7/2 peak corresponding to Au(III) at 86.7 eV and another peak at 92.6 eV which could be attributed to metallic iron 3s due to contamination of the sample from the stainless‐steel sample holders.

Figure 7c,d showed the Au 4f region for sample AuL1NPs/TiO_2_ before and after the reaction. The shape of the peaks showed the co‐existence of more than one gold species, which is why two doublets have been introduced. Focusing the attention on the peak corresponding to 4f7/2, the position of the contributions that conform it, 82.9 and 85 eV, confirmed the existence of gold in two oxidation states, being Au(0) and Au(I) respectively,^[^ ^41^, ^42^, ^43^ ^]^ as expected. When analyzing the sample post‐reaction, no major changes were observed, the main oxidation state was metallic gold with its 4f7/2 peak located at 83.1 eV. Peaks at 84.7 and 86.1 eV can also be observed which were assigned as Au(I) and Au(III), respectively. As in the previous case, an extra peak was seen at 92.4 eV which was attributed to the 3s orbital of the metallic iron due to contamination from the sample holders.

Hence, the obtained results from UV–vis spectroscopy and HRTEM measurements confirmed the presence of gold plasmonic nanoparticles for both AuL1a/TiO_2_ and AuL1NPs/TiO_2_ samples, and XPS spectra revealed their main metallic nature. Thus, Au(0) could be attributed as the main active oxidation state in samples with Au(0)‐based co‐catalysts and also for the Au(I) single‐atom based co‐catalysts samples, which partially evolved to Au(0) during the photocatalytic reaction.

Au/Ti and C/Ti ratios were calculated from the XPS data for both samples (Table S1, Supporting Information). In the case of fresh and reacted AuL1NPs/TiO_2_ samples, both the Au/Ti ratio (0.006 vs 0.007, “fresh” vs “after reaction,” respectively) and the C/Ti ratio (0.80 vs 1.00, “fresh” vs “after reaction,” respectively) remained very similar. However, in the case of the fresh and after reaction AuL1a/TiO_2_ samples, the Au/Ti ratio decreased (0.016 vs 0.010 “fresh” vs “reacted,” respectively) after the photocatalytic reaction, which could be explained by the reduction of Au(I) atoms and the consequent formation of Au nanoparticles (see Figure 7), causing the Au species to be less dispersed throughout the surface of the sample. The C/Ti ratio for the AuL1a/TiO_2_ sample slightly increased after the reaction (1.06 vs 1.55), an effect that could be attributed to some ligands bonded with gold being unbonded upon Au reduction, thus causing the unbonded carbon species to be more dispersed throughout the surface of the sample. These results are consistent with the change in the coordination environment of the samples suggested by the HRTEM observations and the observed induction period attributed to a reorganization of the Au‐containing TiO_2_ hybrid samples during the photocatalytic reaction.

**Scheme 1:**
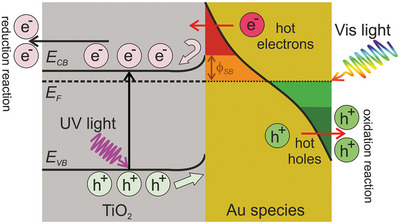
Simplified energy band distribution diagram for the studied hybrid Au/TiO_2_ photocatalysts in this work. E_F_, Fermi energy; E_CB_, energy of conduction band; E_VB_, energy of valence band, *ϕ_SB_* Schottky barrier.

Therefore, the use of different Au species in combination with TiO_2_ achieved several key points that boosted their photoactivity: I) the catalytic character of Au species induced an enhancement of the H_2_ evolution reaction; II) due to the nature of the Au species tested, plasmonic optical resonances under visible light irradiation could generate high energy electrons (hot electrons); III) when TiO_2_ came into contact with Au species, producing the hybrid Au/TiO_2_ photocatalysts reported in this study, their Fermi levels (*E_F_*) equilibrated. To do so, a transfer of free electrons from TiO_2_ to Au species occurred, since the TiO_2_ work function value (4.1–4.4 eV)^[^ ^44^ ^]^ is lower than the 5.1–5.3 eV work function value reported for Au species.^[^ ^45^ ^]^ The resultant accumulation of charges occurring at the Au/TiO_2_ heterojunction induced a band bending of the energy bands of the semiconductor that resulted in a Schottky barrier (*ϕ_SB_*), i.e., an energy barrier for electrons; when the hybrid Au/TiO_2_ photocatalysts were exposed to UV–vis irradiation, the system became out of equilibrium and the photogenerated electrons and holes were distributed throughout the band structure of the hybrid Au/TiO_2_ photocatalysts minimizing their energy, as depicted in **Scheme** **1**. According to the obtained results (see Figure 2), all the studied Au(I)‐complexes and Au(0)‐systems accounted as effective cocatalysts for the selective transport of holes and, therefore, decreasing the recombination of charge carriers as a result of the generated Schottky barrier. We hypothesize that the *ϕ_SB_* value could be affected by the Au(0), Au(I), and Au(III) ratios of the hybrid Au/TiO_2_ photocatalysts, leading to the different photocatalytic activities observed in Figure 2.

### 2.4 Stability

**Figure 8:**
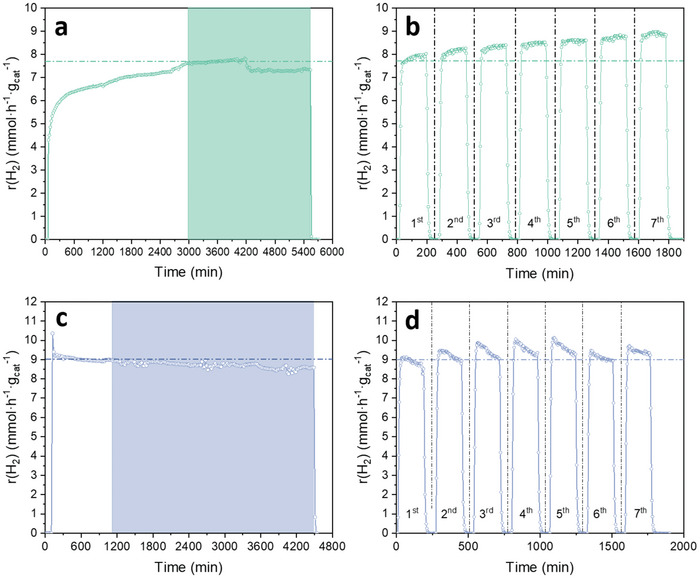
Stability tests. (a,c) Continuous stability test of (a) AuL1a/TiO_2_ (Au 0.32 wt%) and (c) AuL1NPs/TiO_2_ (Au 0.86 wt%) (the highlighted area is considered as the stability test period; see section Photocatalytic Experiments). (b,d) Cyclic stability tests of (b) AuL1a/TiO_2_ (Au 0.32 wt%) and (d) AuL1NPs/TiO_2_ (Au 0.86 wt%).

Two types of stability tests were carried out, i.e., long‐term and cyclic stability tests, to evaluate the durability of the studied hybrid photocatalysts studied in this work (see section Photocatalytic Experiments). The long‐term stability tests for both AuL1a/TiO_2_ and AuL1NPs/TiO_2_ (**Figure** **8**a,c) showed reasonably stable hydrogen photoproductions, as in both cases the decrease on the hydrogen production rate was less than 5% after running a continuous photocatalytic reaction for more than 40 h. Regarding the cyclic tests to assess the performance of the hybrid photocatalysts in consecutive dark‐UV light irradiation steps, it was observed that after seven consecutive cycles the AuL1a/TiO_2_ sample was not only deactivated but continued increasing its hydrogen production rate to around a 16%, as stated in Figure 8b, comparing the H_2_ production rates of the first and the seventh cycle. A similar behavior was observed for AuL1NPs/TiO_2_ (Figure 8d), in which the hydrogen photoproduction rates increased cycle by cycle until the fifth cycle, where stable values of hydrogen production rates were reached. Therefore, the results on the stability tests demonstrated a high stability of the prepared hybrid photocatalysts under the studied experimental conditions.

## 3 Conclusion

We have demonstrated that all prepared hybrid photocatalysts based on coumarin‐Au(I) complexes and coumarin‐Au(0) systems exhibited higher activities than bare TiO_2_ P90 and Au/TiO_2_ reference samples, concluding that the choice of thiocoumarin based ligands was crucial. Among all samples, AuL1NPs/TiO_2_ and AuL1a/TiO_2_ showed the highest hydrogen photoproduction rates, ca. 2.6 and 2.7 times higher than the hydrogen photoproduction rate of the reference Au/TiO_2_ sample, respectively, under the same gold basis (0.25 wt%). After deep analysis of the differences among the hybrid photocatalysts based on Au(I) complexes and Au (0) systems, we concluded that, in case of Au(I)‐based complexes, the inclusion of the DAPTA group in the complex was preferred over the PPh_3_ group, as the latter could block part of the incident light from reaching the coumarin group and/or could hamper the combined photocatalytic effect of the coumarin group, Au site, and TiO_2_ under light irradiation, causing a shadowing effect on the hybrid photocatalyst. Additionally, a stoichiometric Au to coumarin ratio of 1:1 proved to be more effective toward hydrogen production than increasing the gold content in the Au:coumarin ratio. Moreover, the inclusion of a longer hydrocarbon chain between the coumarin and the Au‐ PPh_3_ group helped to decrease the shadowing effect when a PPh_3_ was present in the Au(I) complex acting as co‐catalyst onto TiO_2_ P90 photocatalyst. However, in case of Au(0)‐based systems, the behavior of the hydrocarbon chains was different, as the inclusion of long hydrocarbon chains between the coumarin and the Au nanoparticle decreased the photocatalytic activity, which again confirmed the positive impact of the coumarin group on the photocatalytic activity. Raman spectra revealed a slight shift of the main peak related to anatase, suggesting a strong interaction between the gold co‐catalysts and the TiO_2_ P90. The characterization of AuL1a/TiO_2_ by UV–vis spectroscopy, HRTEM, and XPS demonstrated the appearance of surface plasmon resonance bands attributed to gold species and the formation of ca. 3 nm gold nanoparticles during the photocatalytic reaction, indicating the reduction of Au(I)‐based complexes to form metallic gold nanoparticles. The decrease of the obtained bandgap values from the Tauc plots for the hybrid photocatalysts after reaction suggested an enhanced light absorption in the visible spectrum due to the localized surface plasmon resonance effect. Thus, Au(0) species could be attributed as the main photoactive oxidation state in the studied hybrid Au/TiO_2_ samples, since the Au(I) complexes were partially reduced to Au(0) during the photocatalytic H_2_ evolution reaction. The incorporation of coumarin‐based Au(I) complexes and Au(0) systems led to enhancements ≈25% of the hydrogen photoproduction under both visible and UV light irradiation, due to the presence of plasmonic gold nanoparticles. From the obtained results and characterization, we conjecture that the Schottky barrier formed in the Au/TiO_2_ heterojunction could be affected by the particular composition of Au(0), Au(I), and Au(III) species found in each hybrid Au/TiO_2_ photocatalyst, which led to distinct photocatalytic activities. The findings in this work proved that thiocoumarin‐based Au(I) complexes can be tailored to boost the light‐harvesting properties of hybrid photocatalysts, in order to enhance the AQY values of upcoming photocatalytic applications.

## 4 Experimental Section

### Reagents

10‐bromo‐1‐decene (Aldrich), thioacetic acid (Aldrich), Azobisisobutyronitrile (AIBN)(Aldrich), 4‐hydroxycoumarin (Aldrich), hydrochloric acid (Panreac), Toluene (Scharlau), dichloromethane (Scharlau), sodium chloride (Panreac), sodium hydrogencarbonate (Probus), hexane (Scharlau), dimethylformamide (Merck), Potassium carbonate (Probus), methanol (Labkem), Sodium methoxide, diethyl ether (Scharlav), Potassium hydroxide (Panreac), tetraoctylammonium bromide (TOAB) (Aldrich), hydrogen tetrachloroaurate (III) (Johnson Matthey), 1‐hexanethiol (Aldrich), sodium borohydride (SDS), Gold (III) acetate (Alfa Aesar, 99.9%), TiO_2_ P90 (Degussa, ca. 90 m^2^ g^−1^), absolute ethanol (Scharlau), glacial acetic acid (Sigma‐Aldrich), were used without further purification. Milli‐Q water (H_2_O) was routinely used. The phosphane gold chloride precursors [AuCl(PPh_3_)], [AuCl(DAPTA)] (DAPTA = 3,7‐diacetyl‐ 1,3,7‐triaza‐5‐phosphabicyclo[3.3.1]nonane) were prepared from the reaction of [AuCl(tht)] with the appropriate phosphane, as reported elsewhere.^[^ ^46^ ^]^

### Synthesis of the Ligands L1 and L2

L1 (4‐mercapto‐2H‐chromen‐2‐one) was obtained using a procedure reported elsewhere.^[^ ^47^ ^]^ The synthesis of L2 (4‐((10‐mercaptodecyl)oxy)‐2H‐chromen‐2‐one) was based in a synthetic protocol elsewhere reported^[^ ^48^ ^]^ and it was performed as follows (Scheme S2, Supporting Information). To a solution of commercial 10‐bromo‐1‐decene (0.680 g, 3.10 mmol) and thioacetic acid (1.2 mL, 16.1 mmol) in 25 mL of toluene, AIBN (Azobisisobutyronitrile) was added (0.234 g, 1.45 mmol) and the mixture was heated at 95 °C for 5 h. Then the toluene solvent was removed under vacuum and the resulting orange oil was dissolved in dichloromethane. This solution was first washed with a saturated aqueous solution of sodium chloride (50 mL), second washed with a saturated solution of sodium hydrogencarbonate (50 mL) and the resultant mixture was finally dried with anhydrous sodium sulphate. The dichloromethane solvent was removed under vacuum, and the residue was purified by liquid chromatography on a silica gel column using as eluent a mixture of hexane and dichloromethane 1:1 in volume. (Yellow solid, 72% yield, compound A). In a second step, a Williamson reaction between the bromo‐derivative obtained in the first step and 4‐hydroxycoumarin was performed.^[^ ^49^ ^]^ For that, the formerly obtained compound A (0.611 g, 2.07 mmol) was dissolved in 3 mL of dimethylformamide and was added dropwise to a solution of 4‐hydroxycoumarin (0.342 g, 2.07 mmol) and potassium carbonate (0.600 g, 4.34 mmol) in 4 mL of dimethylformamide. The reaction mixture was stirred at 60 °C for 4 h. Then, 50 mL of dichloromethane were added, and the organics were washed with deionized water. After that, the organic phase was dried, evaporated under reduced pressure almost to dryness, and an excess of ethanol was added to precipitate a white solid, that was filtered and dried (Yield: 70%, 0.544 g, compound B). Finally, to introduce the terminal thiol group to the compound B obtained in the previous step (0.544 g, 1.44 mmol), all the precipitate was dissolved in 35 mL of methanol and 1.7 mL of hydrochloric acid 37% (20.5 mmol) was added. The solution was stirred at 75 °C for 3 h. Then, 50 mL of water was added, and the resultant solution was extracted with dichloromethane (3 × 50 mL). The organic phase was dried with anhydrous sodium sulphate and the solvent was removed under vacuum. The residue was washed with hexane to obtain L2 as a white powder in 61% yield. If needed, L2 can be purified by silica gel chromatography using dichloromethane/methanol 20:1 in volume as eluent.

### Preparation of Au(0) Systems and Au(I) Complexes

**Scheme 2:**
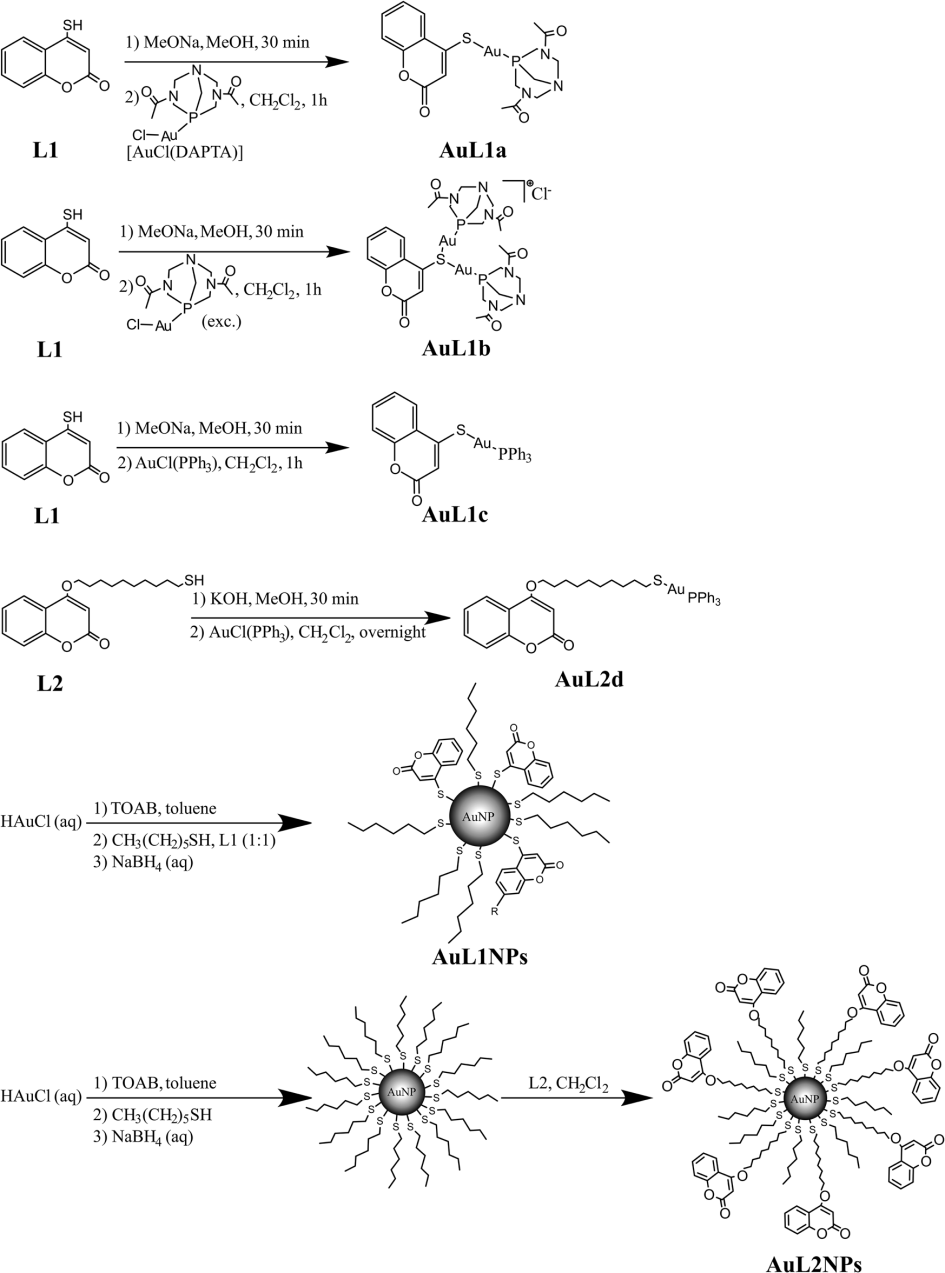
Synthesis of the studied Au(I) complexes and Au(0) systems.

In total, six different gold‐based derivatives (**Scheme** **2**) were prepared. Those could be distinguished into two groups, Au(0) based systems and Au(I) based complexes, each of them with different combinations of organic ligands, being the coumarin the common ligand among all the synthesized complexes. As result of the combination of different ligands, the gold content in the molecular weight of the resultant co‐catalysts varied from 25% to 85%.

### Synthesis of Au(I)‐based Complexes

AuL1a preparation started with the addition of 37 mg of sodium methoxide (0.69 mmol) to a solution of 99 mg of L1 (0.56 mmol) in 10 mL of methanol, and the mixture was stirred for 30 min.^[^ ^21^ ^]^ Then, a solution of 121 mg (0.53 mmol) of [AuCl(DAPTA)] in 10 mL of a mixture 1:1 of dichloromethane and methanol was added and stirred for 1 h. The solution was concentrated under reduced pressure until the solvent was removed and the obtained solid was filtered, washed with diethyl ether, and dried under reduced pressure. 204 mg of [Au(L1)(DAPTA)] were obtained as a white powder (Yield: 61%).

The preparation of AuL1b followed an analogous procedure used for AuL1a but, in this case, 18 mg of L1 (0.108 mmol) and 100 mg of [AuCl(DAPTA)] (0.216 mmol) were used (Yield 78%).

AuL1c was prepared as follows. Sodium methoxide (12 mg, 0.22 mmol) was added to a solution of L1 (33 mg, 0.19 mmol) in 10 mL of methanol, and the mixture was stirred at room temperature for 30 min. Then, a solution of [AuCl(PPh_3_)] (91 mg, 0.18 mmol) in 10 mL of dichloromethane was added and stirred overnight. The solution was concentrated to dryness and the solid obtained was recrystallized in dichloromethane/hexane, filtered, and dried under vacuum (Yield: 60%).

When preparing AuL2d, potassium hydroxide (0.011 g, 0.20 mmol) was added to a solution of L2 (0.033 g, 0.10 mmol) in 10 mL of methanol and the mixture was stirred at room temperature for 30 min. Then, a solution of [AuCl(PPh_3_)] (0.048 g, 0.01 mmol) in dichloromethane was added and the colorless solution reacted overnight protected from light. The solution was evaporated until small volume and diethyl ether were added to precipitate a withe solid, that was filtered and dried (Yield: 71%).

### Synthesis of Au(0)‐based Systems

The preparation of AuL1NPs was made as follows. According to the Burst–Shiffrin method,^[^ ^50^ ^]^ 40 mL of a solution 0.05 m of tetraoctylammonium bromide (TOAB) in toluene (2 mmol) was added to 15 mL of an aqueous solution of hydrogen tetrachloroaurate (III) 0.03 M (0.45 mmol), as stated in Scheme 2. The mixture was vigorously stirred until all the gold was transferred to the organic phase. Then, a mixture of 31 µL of 1‐hexanethiol (0.21 mmol) and 37 mg of L1 (0.21 mmol) in 5 mL of 1:1 toluene/dichloromethane mixture was added. Finally, a freshly prepared aqueous solution of sodium borohydride 0.4 m (12.5 mL, 5 mmol) was slowly added with constant and vigorous stirring. The solution was left stirring for 3 h and then the organic phase was separated and the solvent was evaporated under vacuum. Then, 200 mL of ethanol were added, and the obtained solid was first centrifugated and washed in ethanol (3 × 25 mL) and afterward with a mixture of 10:1 ethanol/hexane (3 × 25 mL). The gold nanoparticles functionalized with 1‐hexanethiol and L1 were obtained as a dark‐greyish‐powder.

To prepare AuL2NPs, a ligand interchange reaction was performed. Thus, in a first step gold nanoparticles functionalized with 1‐hexanethiol were synthesized using the Brust–Schifrin method^[^ ^50^ ^]^ as above mentioned and depicted in Scheme 2. In a second step, 79 mg of these AuNPs were dispersed in 5 mL of dichloromethane, and 36 mg of L2 (0.11 mmol) were added. The mixture was stirred at room temperature for 24 h and the solvent was evaporated to dryness. The residue was washed with ethanol and hexane.

### Preparation of Hybrid Au(I)‐Complex‐TiO_2_ and Au(0) System‐TiO_2_ Photocatalysts

Two series of hybrid Au(I)‐complex‐TiO_2_ and Au(0) system‐TiO_2_ photocatalytic samples were prepared by i) fixing the loading of the co‐catalysts at 1 wt% or by ii) fixing the Au loading at 0.25 wt%. A conventional impregnation method (incipient wetness impregnation, IWI) was used for their preparation. Briefly, dissolution or suspension of each co‐catalyst was prepared in either chloroform for Au(I) based complexes or toluene for Au(0) based systems. The total amount of Au‐based co‐catalyst in each solution/suspension was changed according to the desired mass fraction (wt%) loading over TiO_2_ P90. This dissolution (or suspension) was added dropwise directly on 178.2 mg of TiO_2_ P90 and the resultant impregnated powder was dried in an oven at 50 °C for 3 h. The prepared samples were denoted as AuLXY/TiO_2_, being X the number of the corresponding coumarin ligand (L1 or L2) and Y refers to the group of co‐catalyst, being a, b, c, and d for the gold(I)‐based complexes and NPs for the gold(0)‐based.

### Preparation of Au/TiO_2_ Reference

Two reference samples with different Au loading were prepared by IWI using gold(III) acetate as metal precursor. The required amount of gold(III) acetate was dissolved in 15 mL of glacial acetic acid applying ultrasounds and heat and the resultant solution was added dropwise onto 285 mg of TiO_2_ P90. The samples were first dried at 50 °C for 3 h and then calcined at 300 °C for 3 h, to ensure a strong metal‐support interaction.^[^ ^51^ ^]^ The samples were labeled as Au‐X/TiO_2_, where X refers to the total gold loading in mass fraction (wt%).

### Photocatalytic Experiments

An exact amount of photocatalyst was weighed and dispersed in 1 mL of ethanol to obtain a homogeneous suspension. Conventional laboratory filter paper (F4573 grade, 73gr m^−2^, CHMLAB Group, 170 µm thickness) was used as a fixed bed of the photoreactor. The suspension containing the photocatalyst was impregnated onto a circular zone (diameter 3.9 cm) of the supporting paper. After that, the impregnated paper was dried at 50 °C for 20 min. The photocatalytic experiments were performed under dynamic conditions at room temperature and atmospheric pressure. The photoreactor used in this experiment consists of two cylindrical parts made of glass, where the upper glass part contains a thermocouple, which allows to measure the temperature change of the photocatalytic samples during the experiments (Figure S39, Supporting Information). In this experimental setup, argon (Ar) was used as carrier gas, which was regulated to 20 mL min^−1^ (GHSV = 5900 h^−1^) with a mass‐flow controller (MFC D‐5111, Mass‐Stream M+W Instruments). The Ar_(g)_ was flowed through a Dreschel bottle that contained a water and ethanol mixture in a molar proportion of 9:1. The outlet of the Dreschel bottle was connected to the lower part of the photoreactor, which contains the light source. The photocatalytic sample was placed in the middle of the two parts, with the impregnated side facing the light source. The photoreactor was sealed using an O‐ring made of polydimethylsiloxane (PDMS) and a 3D‐printed clamp. The sample was irradiated with a 4‐+LED light source able to irradiate both UVA (365 nm, 80 mW cm^−2^) and visible light (CCT 6000K). The gas outlet of the photoreactor was connected to a micro gas chromatography (Micro GC 490‐PRO, Agilent) equipped with MS 5 Å, Plot U and Stabilwax columns, which allowed to continuously monitor the outlet gases from the photoreactor. The apparent quantum yield (AQY) was calculated by the following Equation (1):

(1) $$AQY=\frac{2n_{H_{2}}}{n_{p}}\cdot 100=\frac{2nN_{A}}{E_{T}/E_{P}}\cdot 100=\frac{2nN_{A}}{\left(PSt\right)/\left(\frac{h\cdot c}{\lambda }\right)\;}\cdot 100$$

where $n_{H_{2}}$ refers to the number of hydrogen molecules produced and *n_p_* refers to the incident photons reaching the photocatalyst. The number of hydrogen molecules can be calculated as $n_{H_{2}}=nN_{A}$, where *n* was the amount of hydrogen moles produced during a determined time (*t*) of light exposure, and *N_A_* was the Avogadro's number. The *n_p_* can be calculated as *n_p_* = *E_T_* /*E_P_*, where *E_T_* and *E_P_* were the total energy reaching the catalyst and the energy amount of a photon, respectively. The total energy that reaches the catalyst can be expressed as *E_T_* =  *PSt*, where *P* refers to the power density of incident monochromatic light (W m^−2^), *S* was the irradiated area (m^2^) and *t* was the irradiated time (s). *E_P_* can be calculated as *E_P_* =  *hc*/λ, where *h* was the Planck's constant (J·s), *c* was the speed of light (m s^−1^) and λ was the wavelength of the incident light (m).

The photocatalytic experiments were divided into several steps with different combinations of light irradiation (Scheme S1, Supporting Information). The first one was carried out in the absence of light in order to purge the remaining oxygen in the photoreactor. In the second step, ultraviolet (UV) light was turned on until the stabilization of hydrogen production. Once a steady state was reached, visible light irradiation was added to UV (third step) in order to analyze any possible enhancement on hydrogen production values. The addition of visible light resulted in an increase of the sample temperature of ≈5 °C, due to residual heat emitted by the lamp. Once again, this third step was maintained until the hydrogen production reached the steady state. The fourth step consisted of suppressing visible light and keeping only UV light irradiation to check any behavior modification of the sample in comparison to the second step. After reaching the steady state, the fifth step consisted on irradiating UV light while a hot air gun was employed as an external heat source to reach the same temperature value obtained in the third step (combination of both lights). As it was known that visible light can contribute to the enhancement of photocatalytic activity due to thermal promotion,^[^ ^52^ ^]^ this step allowed to separate the contribution of heat to the enhancement of hydrogen photoproduction by visible light. After reaching the steady state, the system was left to cool down to its previous temperature under UV light irradiation and wait till the H_2_ production was again stabilized, to check any further activation of the sample, comparing with steps second and fourth. In the case that a higher activity than step fourth was detected, both UV and Vis lights were again turned on to check any possible higher H_2_ production activity than the observed in the third step. Finally, all the irradiating light sources were turned off and the experiment was concluded.

Two types of stability tests were carried out, on the one hand, long‐term stability tests (UV light irradiation for more than 40 h) and, on the other hand, UV light on/off cyclic stability tests with ca. Three hours light irradiation periods. In the long‐term tests, the starting point was considered as the time at which the sample had reached the steady state of the observed hydrogen production rate. In the case of the cyclic tests, a previous light irradiation step was carried out to reach the steady state of the observed hydrogen production rate, with the aim of avoiding the observation of the induction period during the cyclic stability test. After reaching the steady state on the hydrogen production rate, seven on/off UV light irradiation cycles were carried out, divided into 3 h of light irradiation and 1.5 h of dark conditions in each cycle.

### Characterization of the Au(I) and Au(0) Systems

Infrared spectra have been recorded on an FT‐IR Nicolet iS 5 Spectrophotometer. ^1^H NMR (δ(TMS) = 0.0 ppm) and ^31^P{^1^H} NMR (δ(85% H_3_PO_4_) = 0.0 ppm) spectra were recorded at 400 or 500 MHz using Varian and Bruker spectrometers at 25 °C. ESI mass spectra were recorded on a Fisons VG Quatro spectrometer. Thermogravimetric Analysis (TGA) was performed on an IGA 851 Mettler‐Toledo instrument, nitrogen flow (50 mL min^−1^), from 30 to 1000 °C at a gradient of 10 °C min^−1^. Transmission electron microscopy (TEM) images were registered at 200 kV using a JEOL 2010F instrument having a point resolution of 0.21 nm.

### Characterization of Hybrid Catalysts

Raman spectroscopy was carried out on a confocal Raman spectrometer (Renishaw in Via Qontor) equipped with a Leica DM2700 M micro‐scope over the range of 50–800 cm^−1^. A laser excitation source of 532 nm and a grating of 2400 lines mm^−1^ were used. A power of the laser of 1 mW·cm^−2^ was kept in all samples. UV–vis reflectance spectroscopy was measured on a double beam spectrophotometer Shimadzu UV2700i UV–vis/NIR equipped with an ISR 3100 Ulbricht integrating sphere. BaSO_4_ was used as a reference standard. The spectra were recorded at room temperature in air atmosphere within the range of 300–800 nm. The acquired diffuse reflectance spectra were converted to absorbance through the standard Kubelka–Munk function *F(R)*.^[^ ^53^ ^]^ The band gap energies (E_g_) of the prepared samples were estimated from the UV–vis spectra by Tauc plots of [*F(R)* hν]^1/2^ versus hν, using a modified version of the Tauc's equation,^[^ ^54^, ^55^ ^]^ where the material absorption coefficient (α) can be substituted by *F(R)*, since they were proportional to each other.^[^ ^56^ ^]^ High‐resolution transmission electron microscopy (HRTEM) and high‐angle annular dark‐field scanning transmission electron microscopy (HAADF‐STEM) were carried out on a FEI Tecnai F20 electron microscope equipped with a field emission electron gun and operated at an accelerating voltage of 200 kV. X‐ray photoelectron spectroscopy (XPS) was performed using a SPECS system equipped with a PHOIBOS 150 EP hemispherical energy analyser, an MCD‐9 detector, and an XR‐50 X‐ray source operating at 150 W. CasaXPS program (Casa Software Ltd., UK) was used to analyze the XPS data (Shirley type background).

## Conflict of Interest

The authors declare no conflict of interest.

## Supporting information



Supporting Information

## Data Availability

The data that support the findings of this study are available from the corresponding author upon reasonable request.

## References

[1] N. Salmon , R. Bañares‐Alcántara , Sustain. Energy Fuels 2021, 5, 2814.

[2] I. Lucentini , X. Garcia , X. Vendrell , J. Llorca , Ind. Eng. Chem. Res. 2021, 60, 18560.

[3] E. Aguiló , L. Soler , A. Casanovas , A. J. Moro , J. C. Lima , L. Rodríguez , J. Llorca , ChemCatChem 2017, 9, 3289.

[4] A. I. Osman , N. Mehta , A. M. Elgarahy , M. Hefny , A. Al‐Hinai , A. H. Al‐Muhtaseb , D. W. Rooney , Environ. Chem. Lett. 2022, 20, 153.

[5] E. B. Agyekum , C. Nutakor , A. M. Agwa , S. Kamel , Membranes (Basel) 2022, 12, 173. 35207094 10.3390/membranes12020173 PMC8880752

[6] R. Kumar , A. Kumar , A. Pal , Mater. Today Proc. 2021, 46, 5353.

[7] I. Dincer , C. Acar , Int. J. Hydrogen Energy 2014, 40, 11094.

[8] M. Ji , J. Wang , J. Wang , Int. J. Hydrogen Energy 2021, 46, 38612.

[9] Y.‐J. Xu , Front. Catal. 2021, 1, 708319.

[10] C. Acar , I. Dincer , G. F. Naterer , Int. J. Energy Res. 2016, 40, 1449.

[11] M. Younas , S. Shafique , A. Hafeez , F. Javed , F. Rehman , Fuel 2022, 316, 123317.

[12] P. Wang , G. Liang , C. E. Webster , X. Zhao , Eur. J. Inorg. Chem. 2020, 2020, 3534.

[13] M. Wang , Y. Na , M. Gorlov , L. Sun , Dalton Trans. 2009, 6458, https://pubs.rsc.org/en/content/articlelanding/2009/dt/b903809d/unauth 19672488 10.1039/b903809d

[14] W. J. Youngblood , S.‐H. A. Lee , K. Maeda , T. E. Mallouk , Acc. Chem. Res. 2009, 42, 1966. 19905000 10.1021/ar9002398

[15] M. Teranishi , T. Kunimoto , S. Naya , H. Kobayashi , H. Tada , J. Phys. Chem. C 2020, 124, 3715.

[16] K. E. Dalle , J. Warnan , J. J. Leung , B. Reuillard , I. S. Karmel , E. Reisner , Chem. Rev. 2019, 119, 2752. 30767519 10.1021/acs.chemrev.8b00392 PMC6396143

[17] M. K. Nazeeruddin , E. Baranoff , M. Grätzel , Sol. Energy 2011, 85, 1172.

[18] M. A. M. Al‐Alwani , A. B. Mohamad , N. A. Ludin , A. A. H. Kadhum , K. Sopian , Renew. Sustain. Energy Rev. 2016, 65, 183.

[19] S. Ardo , G. J. Meyer , Chem. Soc. Rev. 2009, 38, 115. 19088971 10.1039/b804321n

[20] X. Kong , Z. Gao , Y. Gong , H. Huang , H. Wang , P. Liu , H. Yin , Z. Cui , Z. Li , Y. Liang , S. Zhu , Y. Huang , X. Yang , J. Mater. Chem. A Mater. 2019, 7, 3797.

[21] X. Yao , Q. Zhang , P.‐Y. Ho , S.‐C. Yiu , S. Suramitr , S. Hannongbua , C.‐L. Ho , Inorganics (Basel) 2023, 11, 110.

[22] A. Galushchinskiy , R. González‐Gómez , K. McCarthy , P. Farràs , A. Savateev , Energy Fuels 2022, 36, 4625. 35558990 10.1021/acs.energyfuels.2c00178 PMC9082502

[23] J. H. Kim , D. Hansora , P. Sharma , J. W. Jang , J. S. Lee , Chem. Soc. Rev. 2019, 48, 1908. 30855624 10.1039/c8cs00699g

[24] A. G. Mahmoud , M. F. C. Guedes da Silva , A. J. L. Pombeiro , Coord. Chem. Rev. 2021, 429, 213614.

[25] M. Friederici , I. Angurell , M. Seco , O. Rossell , J. Llorca , Dalton Trans. 2011, 40, 7934. 21725529 10.1039/c1dt10456j

[26] M. Murdoch , G. I. N. Waterhouse , M. A. Nadeem , J. B. Metson , M. A. Keane , R. F. Howe , J. Llorca , H. Idriss , Nat. Chem. 2011, 3, 489. 21602866 10.1038/nchem.1048

[27] K. Takanabe , ACS Catal. 2017, 7, 8006.

[28] T. S. Dörr , L. Deilmann , G. Haselmann , A. Cherevan , P. Zhang , P. Blaha , P. W. de Oliveira , T. Kraus , D. Eder , Adv. Energy Mater. 2018, 8, 201802566.

[29] K. Baba , S. Bulou , M. Quesada‐Gonzalez , S. Bonot , D. Collard , N. D. Boscher , P. Choquet , ACS Appl. Mater. Interfaces 2017, 9, 41200. 28990763 10.1021/acsami.7b10904

[30] Y. Li , H. Wang , Q. Feng , G. Zhou , Z. S. Wang , Energy Environ. Sci. 2013, 6, 2156.

[31] Z. Zhang , J. T. Yates , Chem. Rev. 2012, 112, 5520. 22783915 10.1021/cr3000626

[32] X. Huang , M. A. El‐Sayed , J. Adv. Res. 2010, 1, 13.

[33] L. Martínez , M. Benito , I. Mata , L. Soler , E. Molins , J. Llorca , Sustain. Energy Fuels 2018, 2, 2284.

[34] J. Fang , S.‐W. Cao , Z. Wang , M. M. Shahjamali , S. C. J. Loo , J. Barber , C. Xue , Int. J. Hydrogen Energy 2012, 37, 17853.

[35] F. Conte , I. Rossetti , G. Ramis , C. Vaulot , S. Hajjar‐Garreau , S. Bennici , Materials 2022, 15, 2915. 35454608 10.3390/ma15082915 PMC9031976

[36] P. Hepperle , A. Herman , B. Khanbabaee , W. Y. Baek , H. Nettelbeck , H. Rabus , Part. Part. Syst. Charact. 2022, 39, 202200070.

[37] S. Caporali , F. Muniz‐Miranda , A. Pedone , M. Muniz‐Miranda , Sensors 2019, 19, 2700. 31208081 10.3390/s19122700 PMC6631783

[38] A. M. Visco , F. Neri , G. Neri , A. Donato , C. Milone , S. Galvagno , Phys. Chem. Chem. Phys. 1999, 1, 2869.

[39] X. Z. Li , F. B. Li , Environ. Sci. Technol. 2001, 35, 2381. 11414049 10.1021/es001752w

[40] B. R. Cuenya , S.‐H. Baeck , T. F. Jaramillo , E. W. McFarland , J. Am. Chem. Soc. 2003, 125, 12928. 14558841 10.1021/ja036468u

[41] Y. Chen , L. Soler , C. Xie , X. Vendrell , J. Serafin , D. Crespo , J. Llorca , Appl. Mater. Today 2020, 21, 100873.

[42] Z. G. Sun , X. S. Li , J. L. Liu , Y. C. Li , B. Zhu , A. M. Zhu , J. Catal. 2019, 375, 380.

[43] A. Naldoni , M. D'Arienzo , M. Altomare , M. Marelli , R. Scotti , F. Morazzoni , E. Selli , V. Dal Santo , Appl. Catal. B 2013, 130, 239.

[44] V. Mansfeldova , M. Zlamalova , H. Tarabkova , P. Janda , M. Vorokhta , L. Piliai , L. Kavan , J. Phys. Chem. C 2021, 125, 1902.

[45] E. Smirnov , M. D. Scanlon , D. Momotenko , H. Vrubel , M. A. Méndez , P. F. Brevet , H. H. Girault , ACS Nano 2014, 8, 9471. 25184343 10.1021/nn503644v

[46] E. Vergara , S. Miranda , F. Mohr , E. Cerrada , E. R. T. Tiekink , P. Romero , A. Mendía , M. Laguna , Eur. J. Inorg. Chem. 2007, 2007, 2926.

[47] S. K. Ghosh , World J. Pharm. Res. 2017, 6, 1634.

[48] F. Gonzàlez De Rivera , I. Angurell , O. Rossell , M. Seco , J. Llorca , J. Organomet. Chem. 2012, 715, 13.

[49] R. J. Ouellette , J. D. Rawn , in Organic Chemistry, 2nd Ed., (Eds.: R. J. Ouellette , J. D. Rawn ), Elsevier Science & Technology, San Diego, CA 2018, pp. 507–536.

[50] M. Brust , M. Walker , D. Bethell , D. J. Schiffrin , R. Whyman , J. Chem. Soc. Chem. Commun. 1994, 801, https://pubs.rsc.org/en/content/articlehtml/1994/c3/c39940000801

[51] H. Tang , Y. Su , B. Zhang , A. F. Lee , M. A. Isaacs , K. Wilson , L. Li , Y. Ren , J. Huang , M. Haruta , B. Qiao , X. Liu , C. Jin , D. Su , J. Wang , T. Zhang , Sci. Adv. 2017, 3, 1700231. 10.1126/sciadv.1700231 PMC5640381 29043293

[52] A. Castedo , A. Casanovas , I. Angurell , L. Soler , J. Llorca , Fuel 2018, 222, 327.

[53] P. Kubleka , F. Munk , Zeitschrift für Technische Physik 1931, 12, 593.

[54] J. Tauc , R. Grigorovici , A. Vancu , Phys. Status Solidi 1966, 15, 627.

[55] J. Tauc , Mater. Res. Bull. 1968, 3, 37.

[56] J. O. Carneiro , S. Azevedo , F. Fernandes , E. Freitas , M. Pereira , C. J. Tavares , S. Lanceros‐Méndez , V. Teixeira , J. Mater. Sci. 2014, 49, 7476.

