# Supplementary material for: Thiocoumarin‐based Au(I) Complexes and Au(0) Systems over TiO2 as Hybrid Photocatalysts for Hydrogen Generation under UV–Vis Light
Source: Adv Sci (Weinh). 2024 Nov 5;11(47):2404969. doi: 10.1002/advs.202404969 (PMC11653606; doi:10.1002/advs.202404969)
Supplement: Supplementary file 1 — Supporting Information [file ADVS-11-2404969-s001.docx]

Supporting information

Thiocoumarin based Au(I) complexes and Au(0) systems over TiO_2_ as hybrid photocatalysts for hydrogen generation under UV-visible light

*Asier Agrelo-Leston^1^, Jordi Llorca^1^*, Elizabeth Martínez^2^, Inmaculada Angurell^2,3^, Laura Rodríguez^2,3^, Lluís Soler^1^**

*^1^ Institute of Energy Technologies, Center for Research in Multiscale Science and Engineering and Department of Chemical Engineering, Universitat Politècnica de Catalunya (UPC), EEBE, Eduard Maristany 10-14, 08019 Barcelona, Spain.*

*^2^* *Departament de Química Inorgànica i Orgànica, Secció Química Inorgànica, Universitat de Barcelona, Martí i Franquès 1, 08028 Barcelona, Spain.*

*^3^ Institut de Nanociència i Nanotecnologia (IN2UB). Universitat de Barcelona, Avda Diagonal 647,*

*08028 Barcelona, Spain.*

**Table of contents**

Supporting figures 2

Supporting schemes. 22

Supporting Table 22

**Supplementary figures**

**
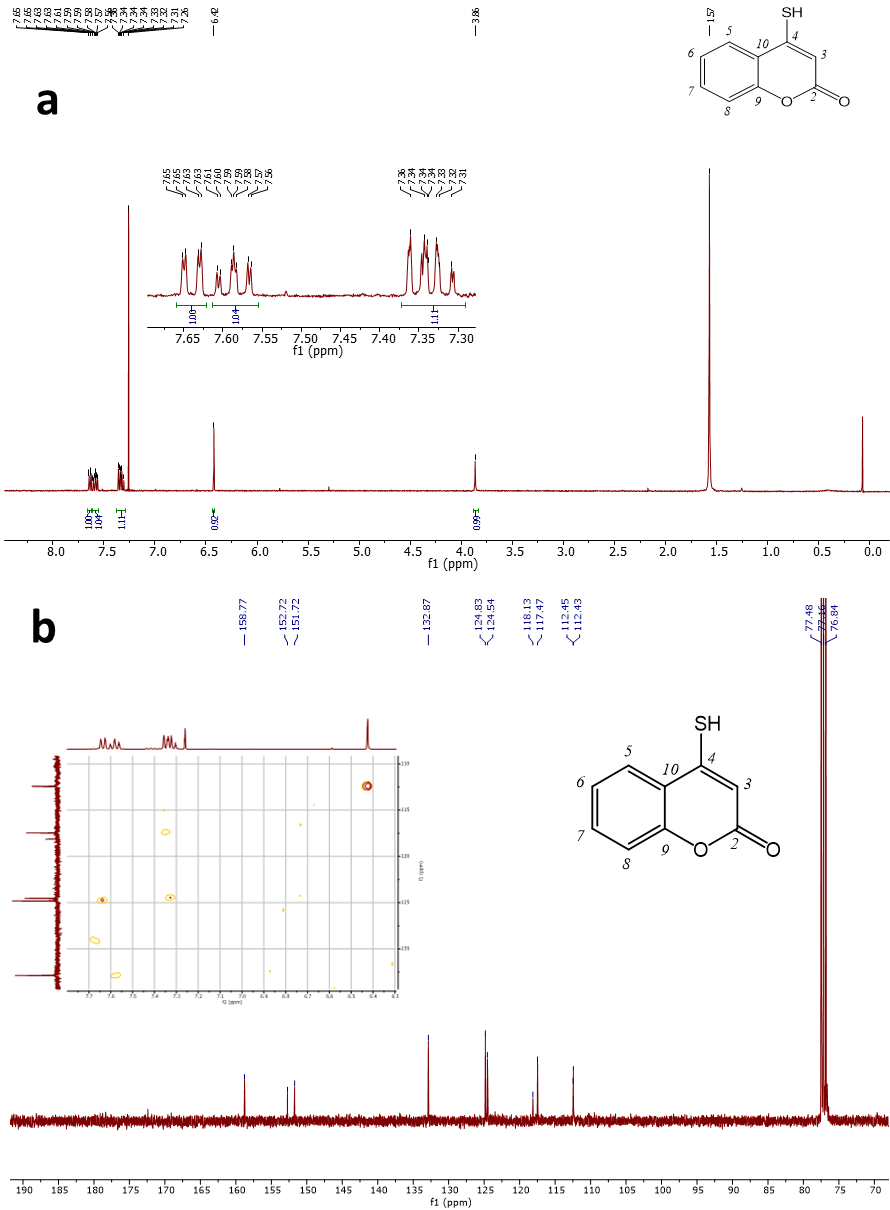
**

**Figure S1**: (a) ^1^H NMR in CDCl_3_ of L1. (b) ^13^C{^1^H} NMR in CDCl_3_ of L1; Inset: HSQC of L1.


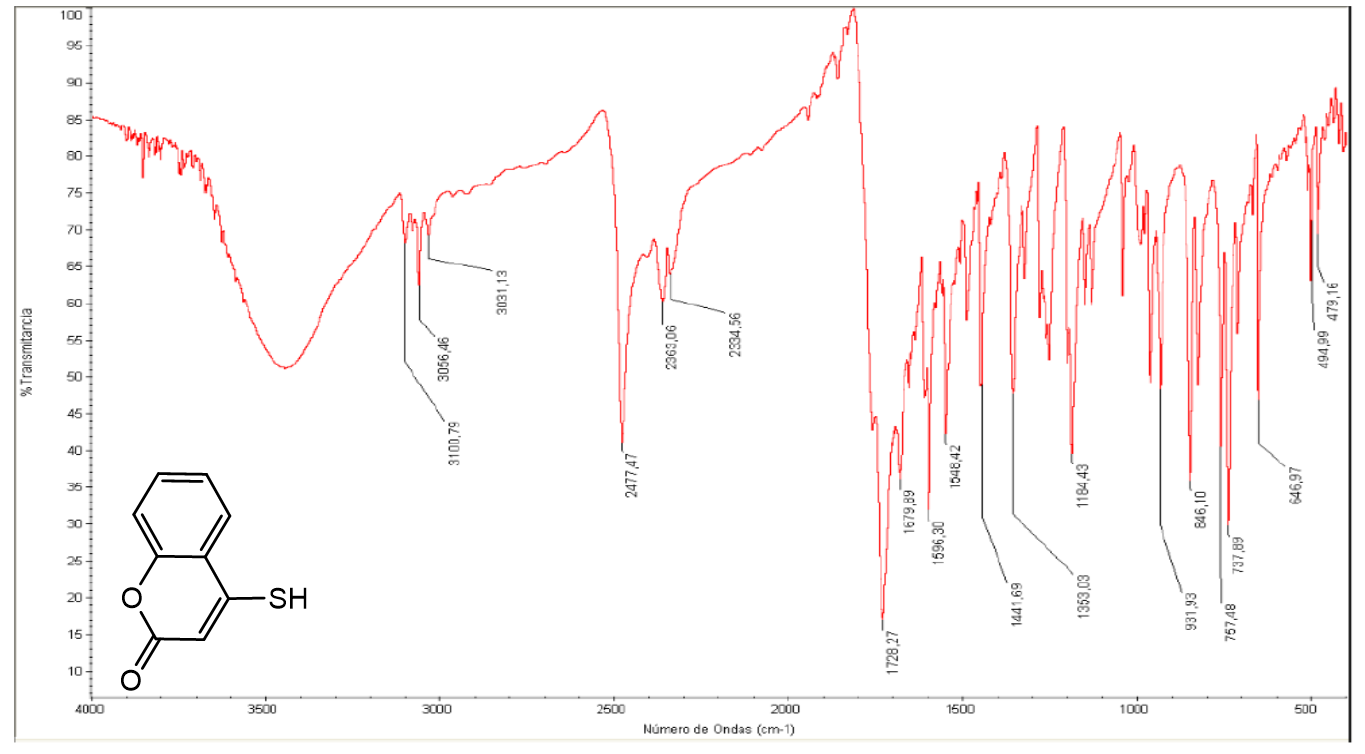


**Figure S2.** IR spectra of L1


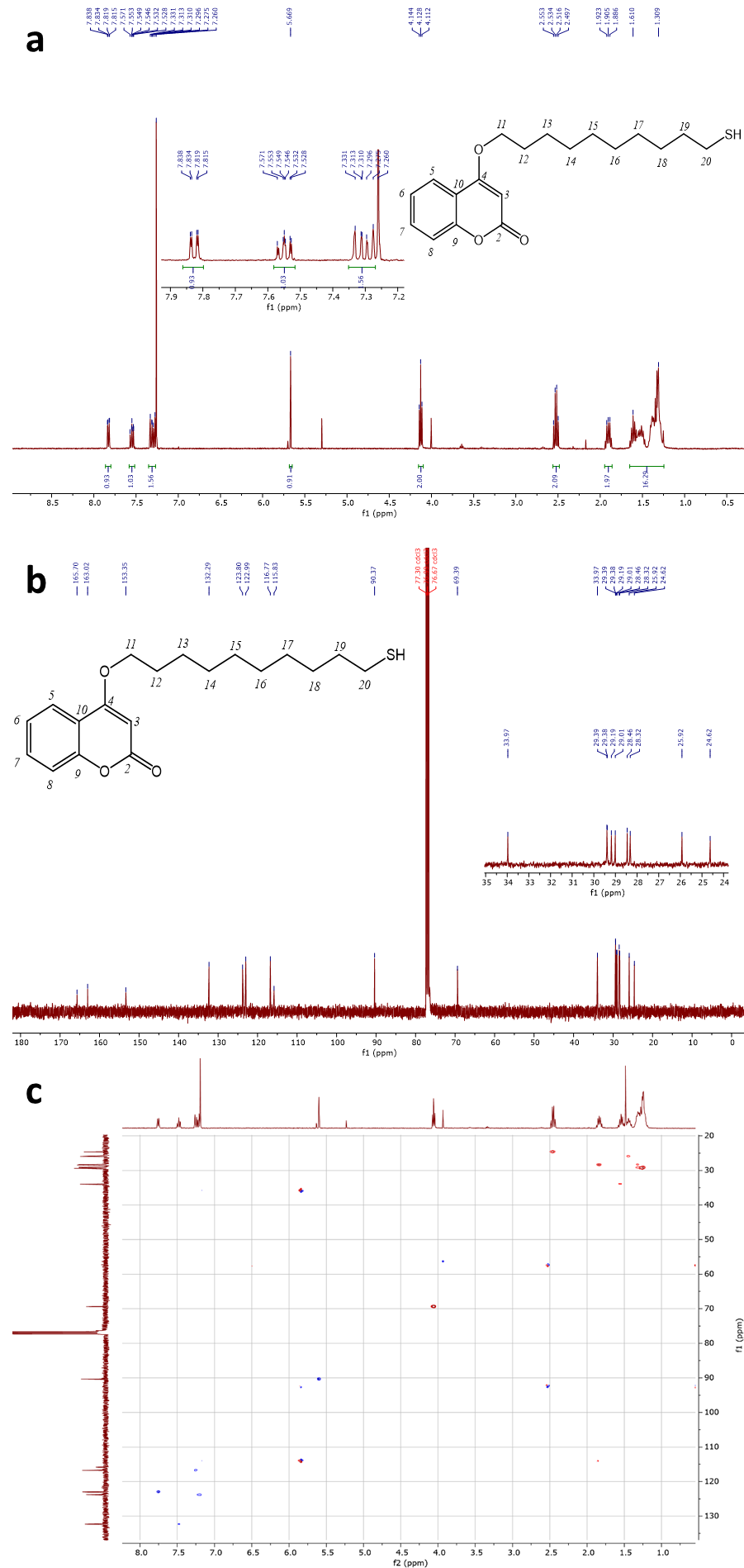


**Figure S3a**: (a) ^1^H NMR in CDCl_3_ of L2. (b) ^13^C{^1^H} NMR and (c) HSQC in CDCl_3_ of L2.


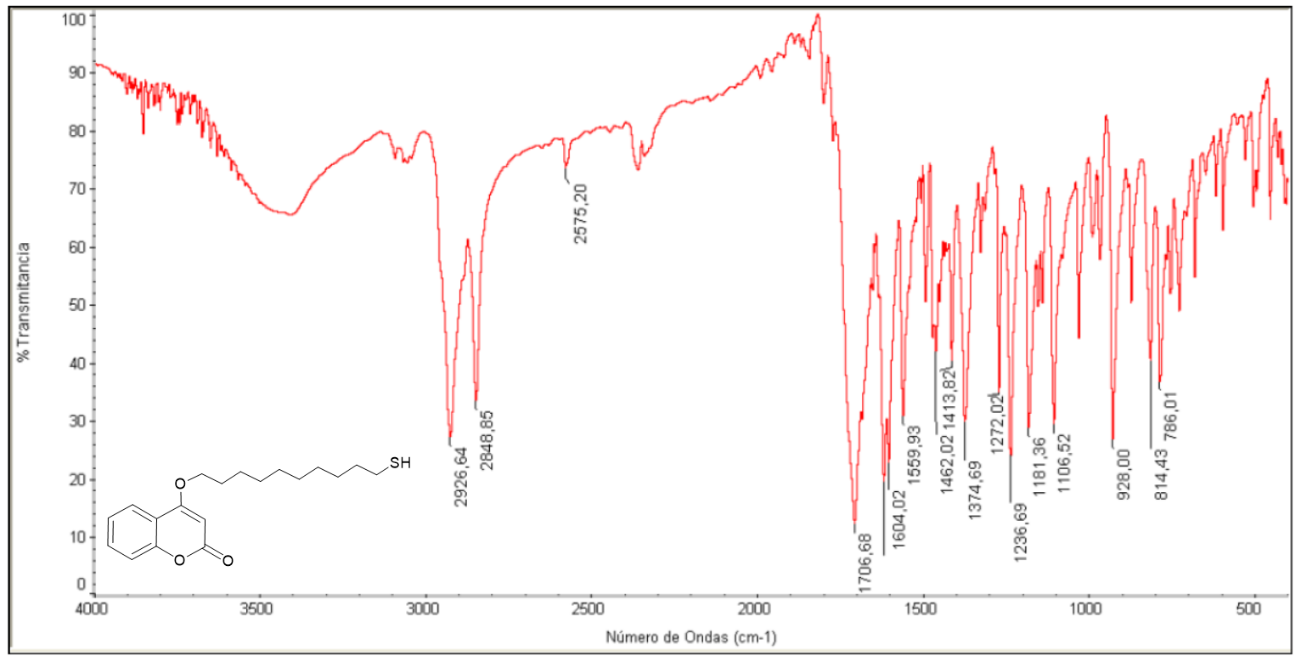


**Figure S4.** IR spectra of L2

**
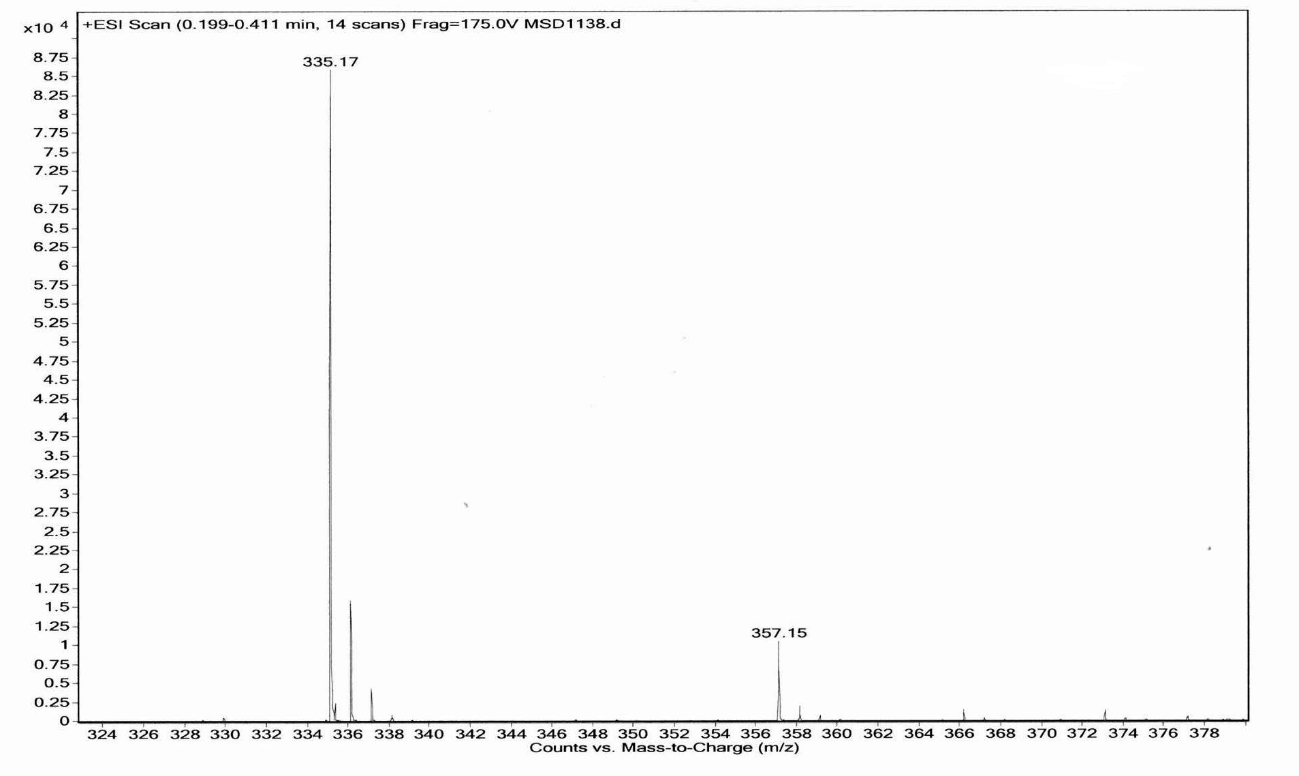
**

[M+Na]^+^

[M+H]^+^

**Figure S5**: ESI(+) MS of **L2**


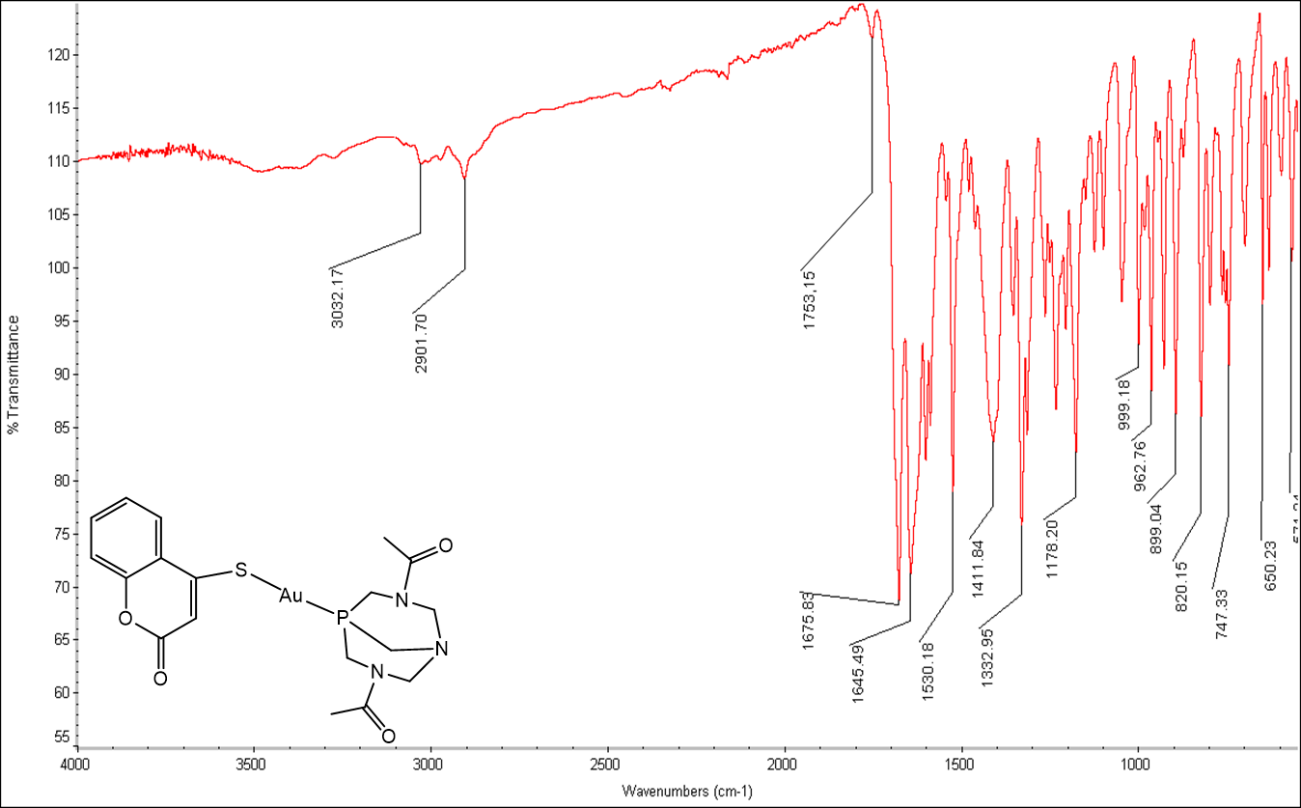


**Figure S6.** IR spectra of AuL1a


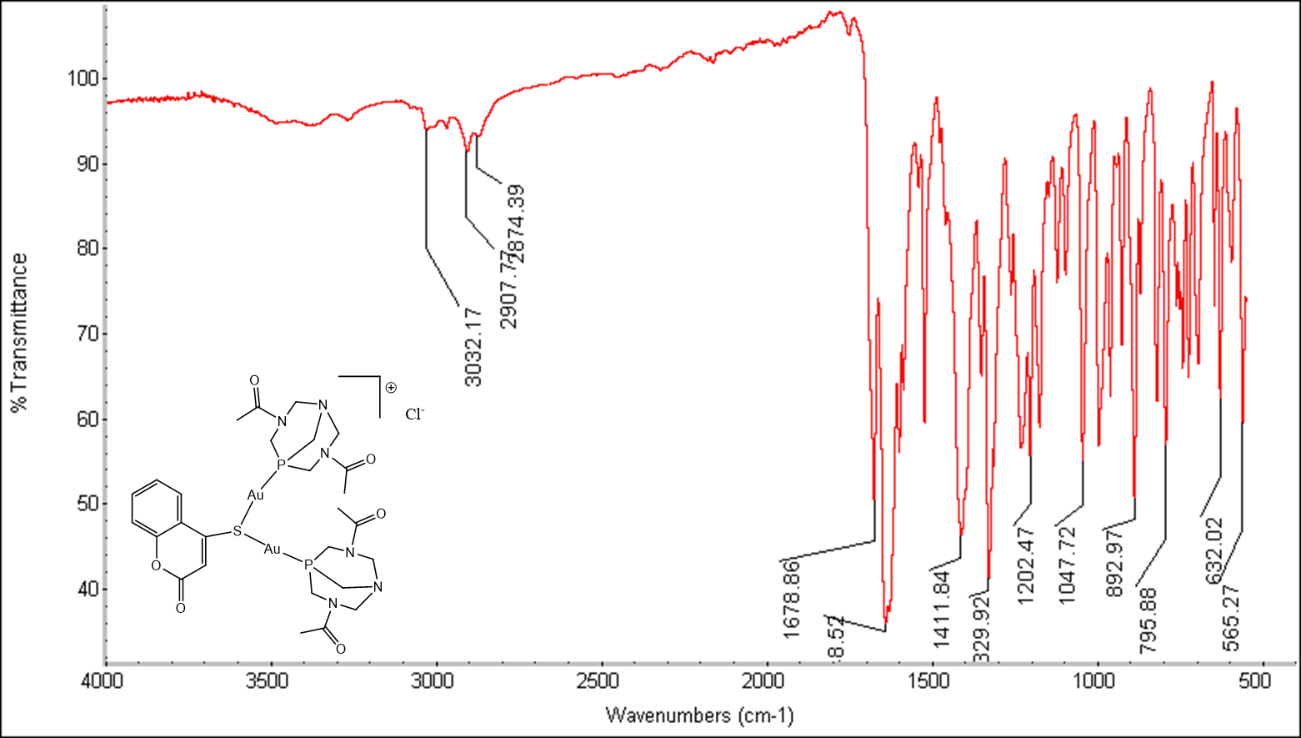


**Figure S7.** IR spectra of AuL1b


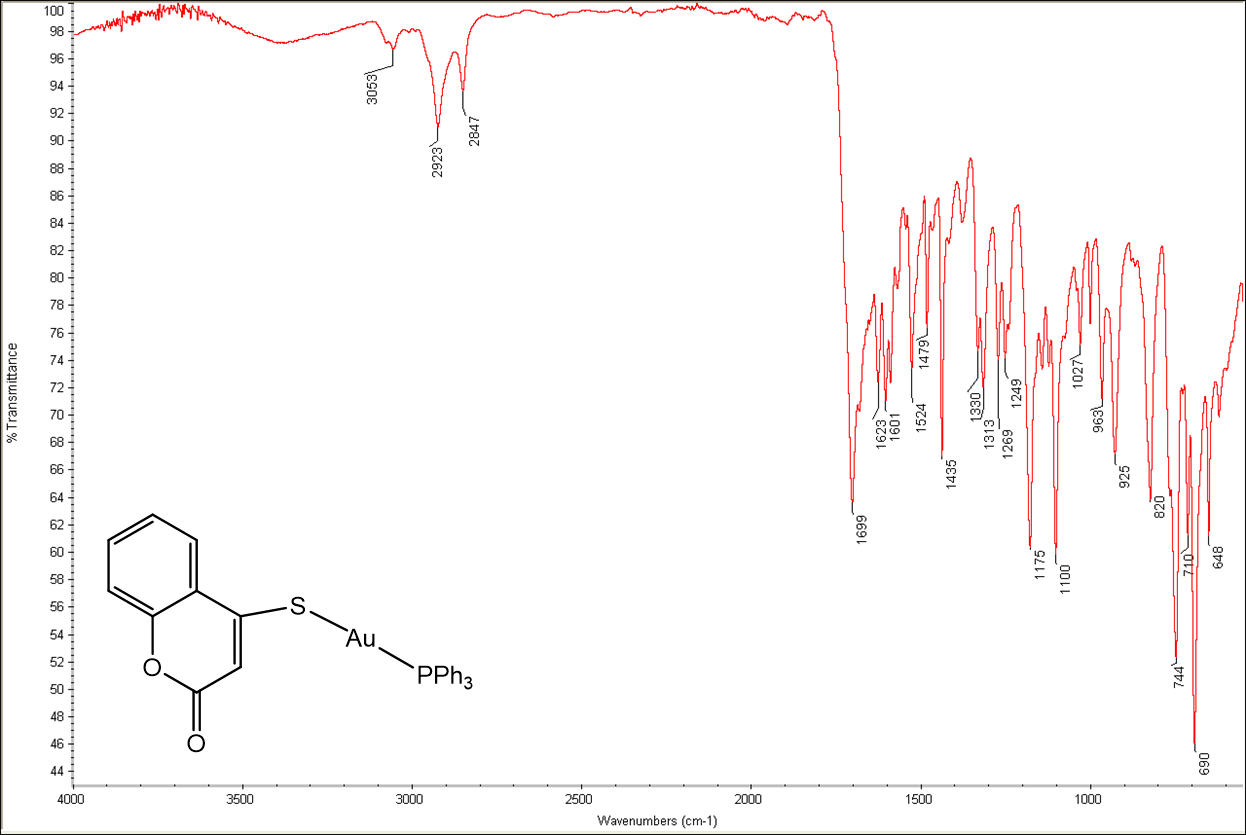


**Figure S8.** IR spectra of AuL1c


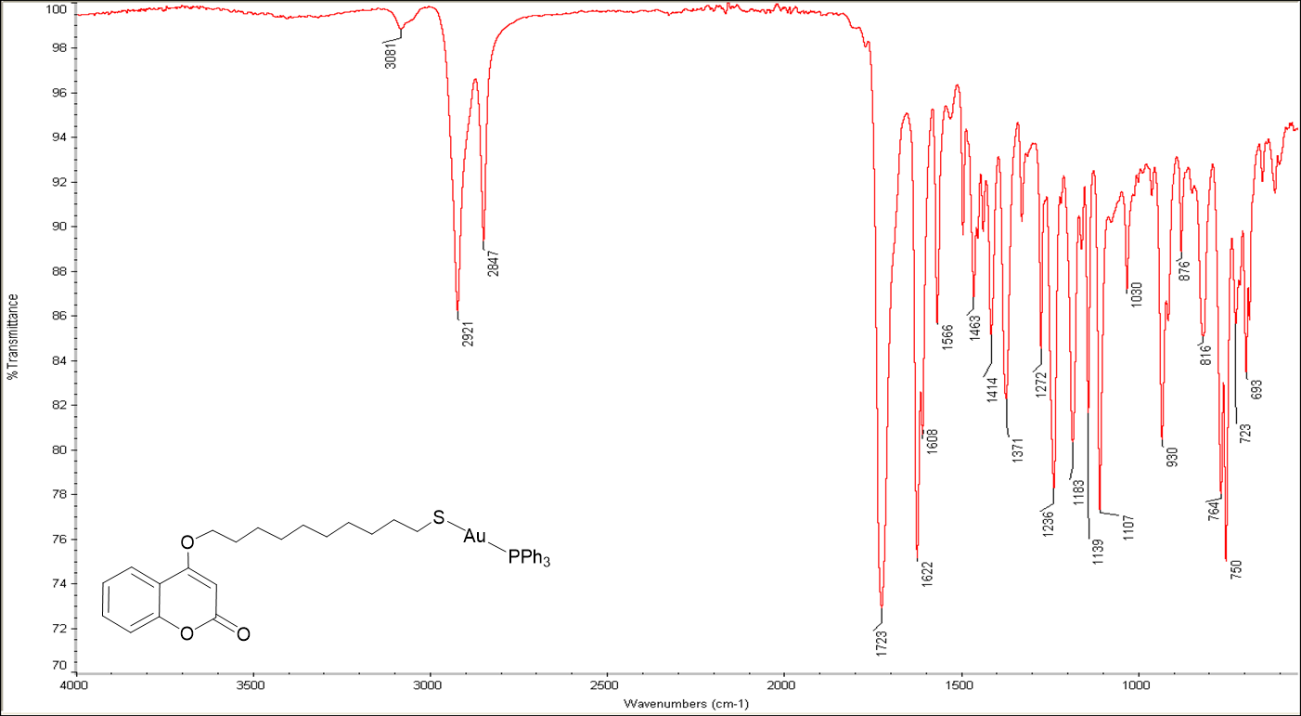


**Figure S9.** IR spectra of AuL2d


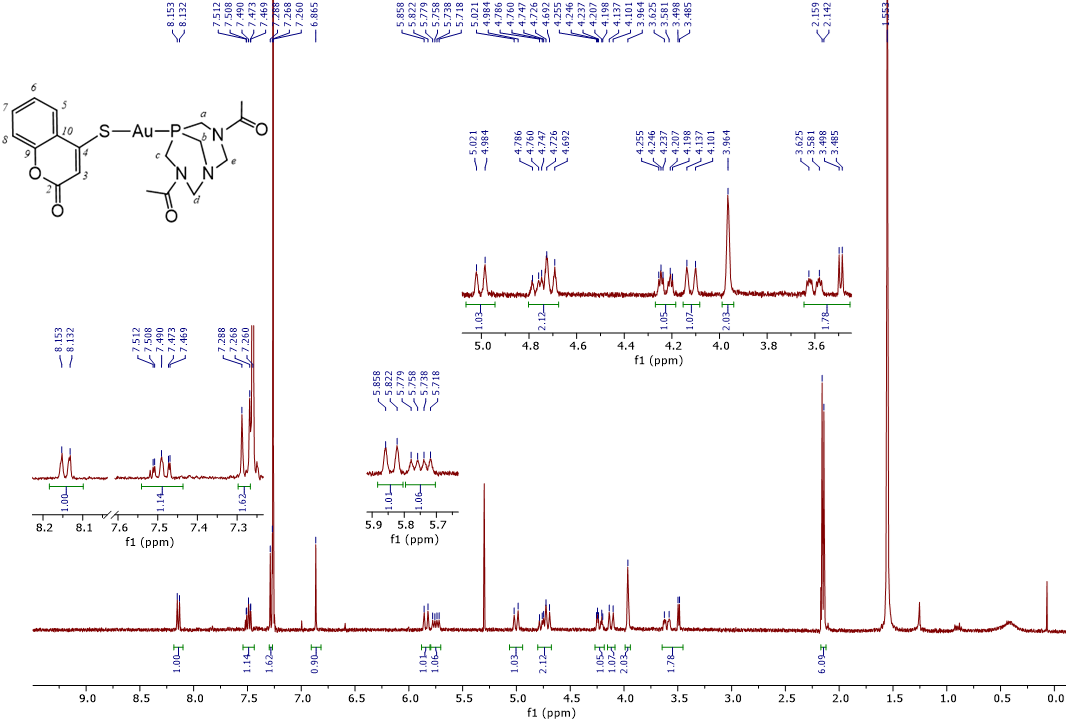


**Figure S10**: ^1^H NMR in CDCl_3_ of AuL1a


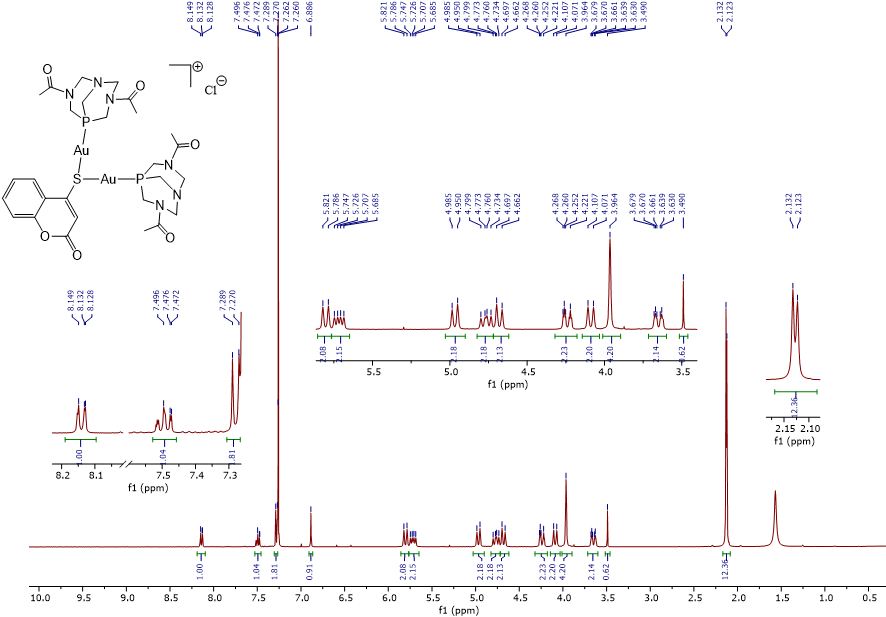


**Figure S11**: ^1^H NMR in CDCl_3_ of AuL1b


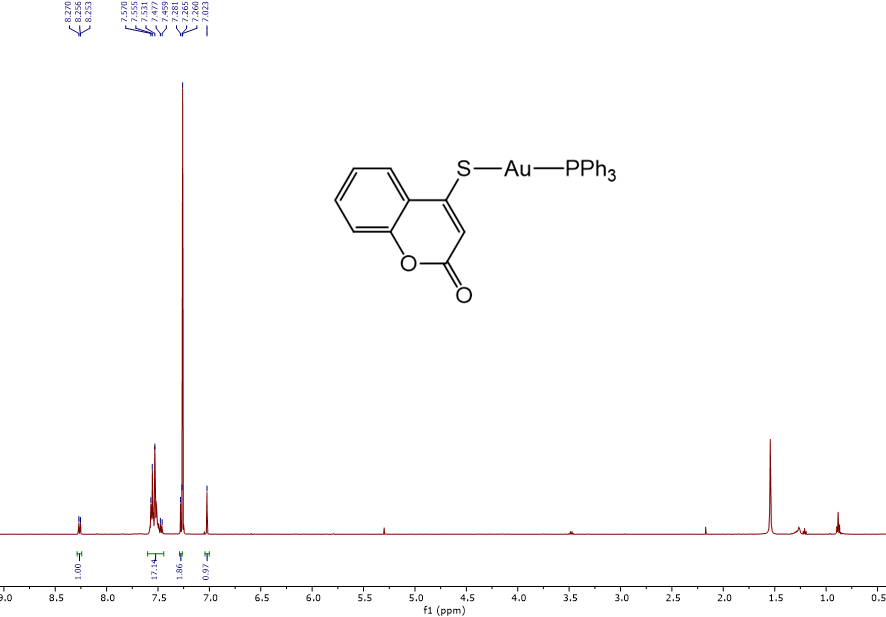


**Figure S12**: ^1^H NMR in CDCl_3_ of AuL1c


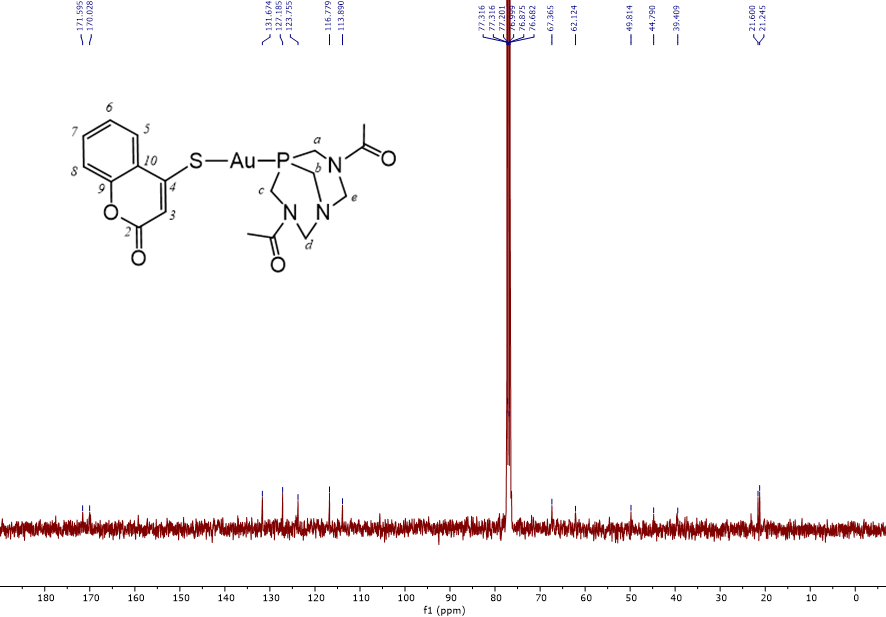


**Figure S13**: ^13^C{^1^H} NMR in CDCl_3_ of AuL1a


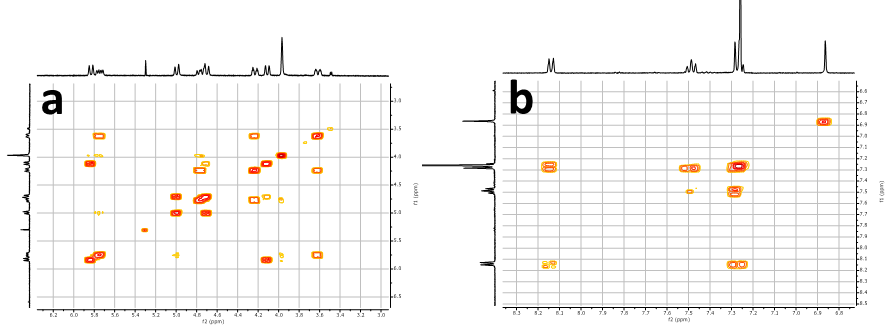


**Figure S14:** COSY (DAPTA signals -a- and coumarin signals -b-) of AuL1a


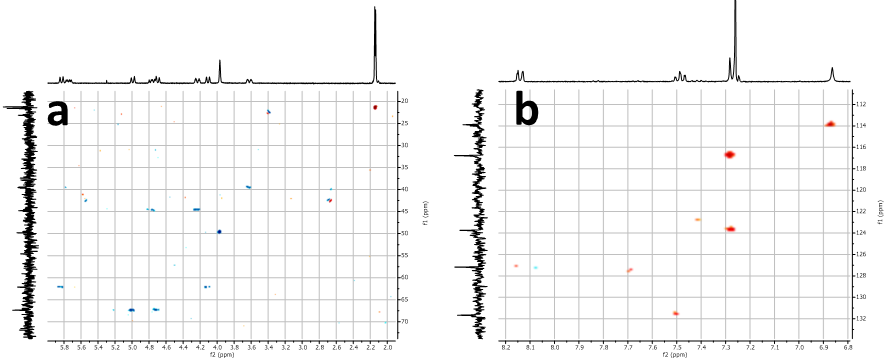


**Figure S15: HSQC** (DAPTA signals -a- and coumarin signals -b-) of AuL1a

**
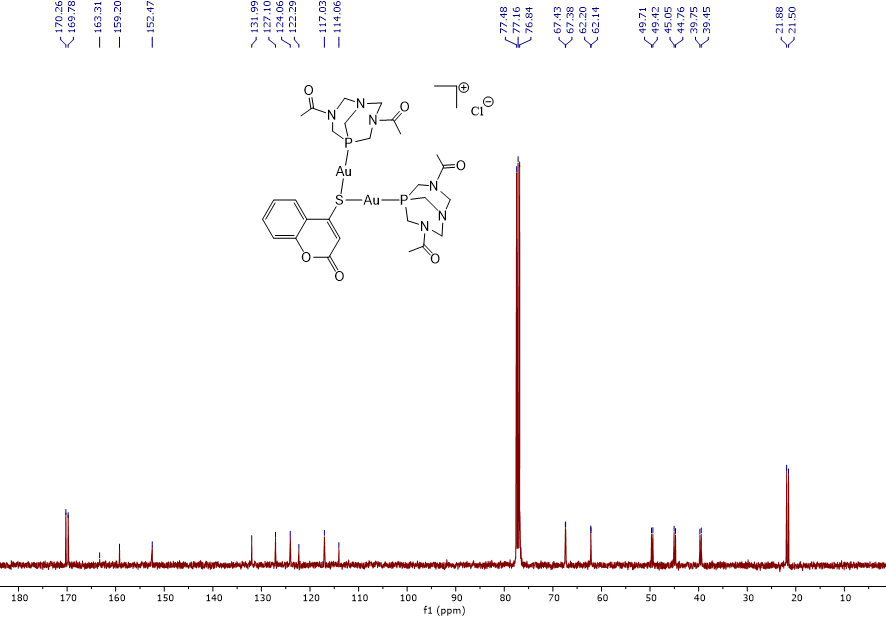
**

**Figure S16**: ^13^C{^1^H} NMR in CDCl_3_ of AuL1b

**
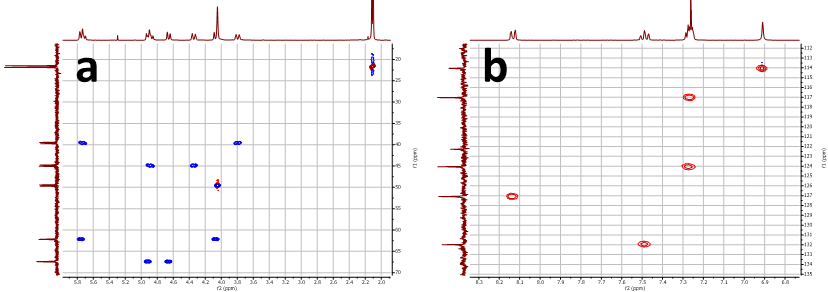
**

**Figure S17**: HSQC of AuL1b ( DAPTA region-a-; coumarin region, -b-)

**Figure S18**: ^31^P{^1^H} NMR in CDCl_3_ of AuL1a

**Figure S19**: ^31^P{^1^H} NMR in CDCl_3_ of AuL1b

**Figure S20**: ^31^P{^1^H} NMR in CDCl_3_ of AuL1c


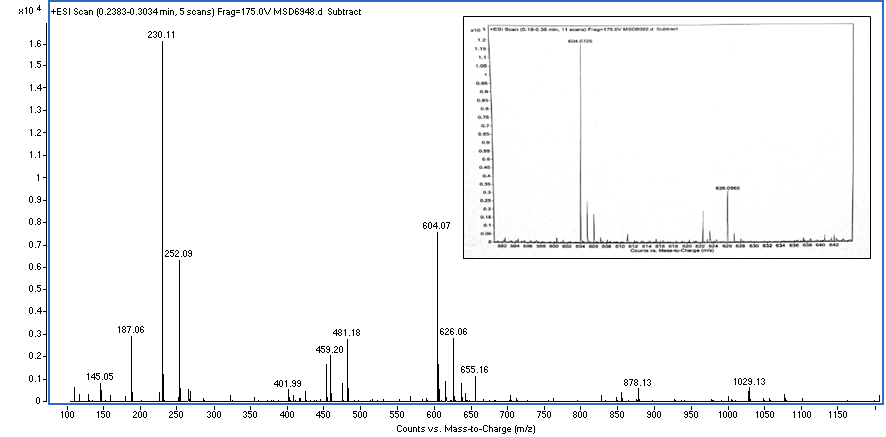


**Figure S21**: ESI(+) MS of AuL1a


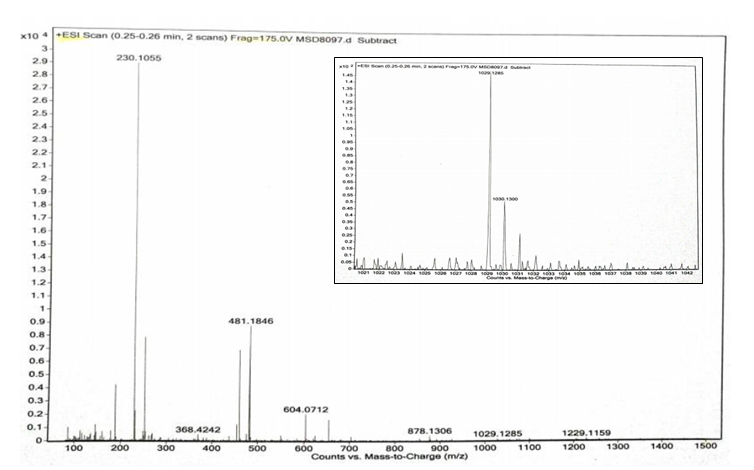


**Figure S22**: ESI MS of AuL1b


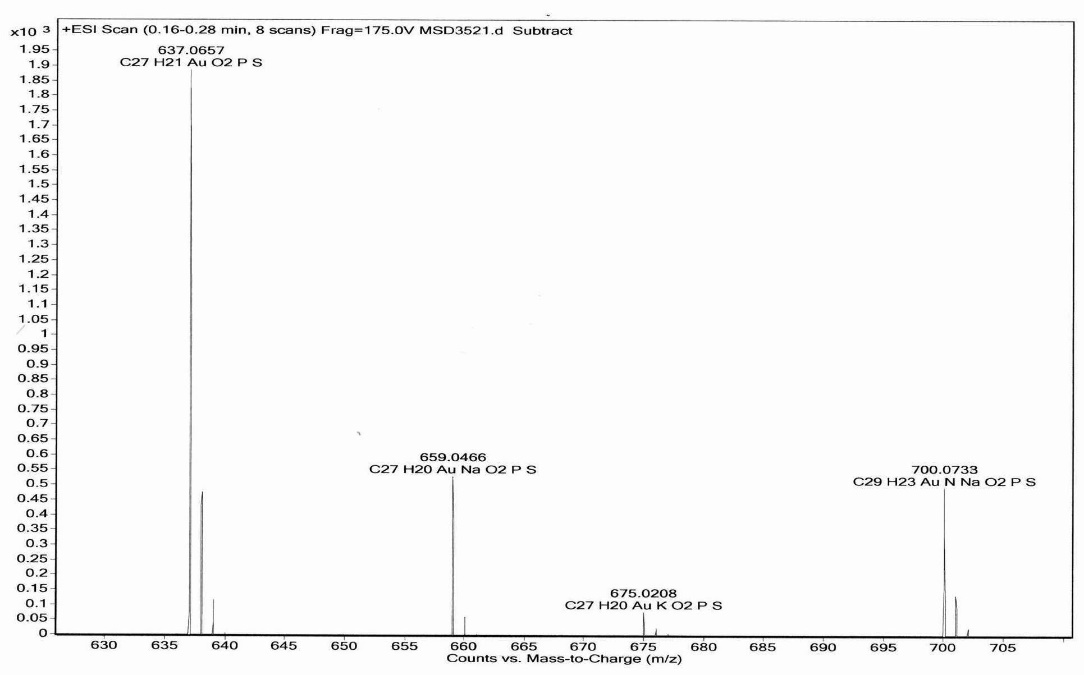


**Figure S23**: ESI(+) MS of AuL1c


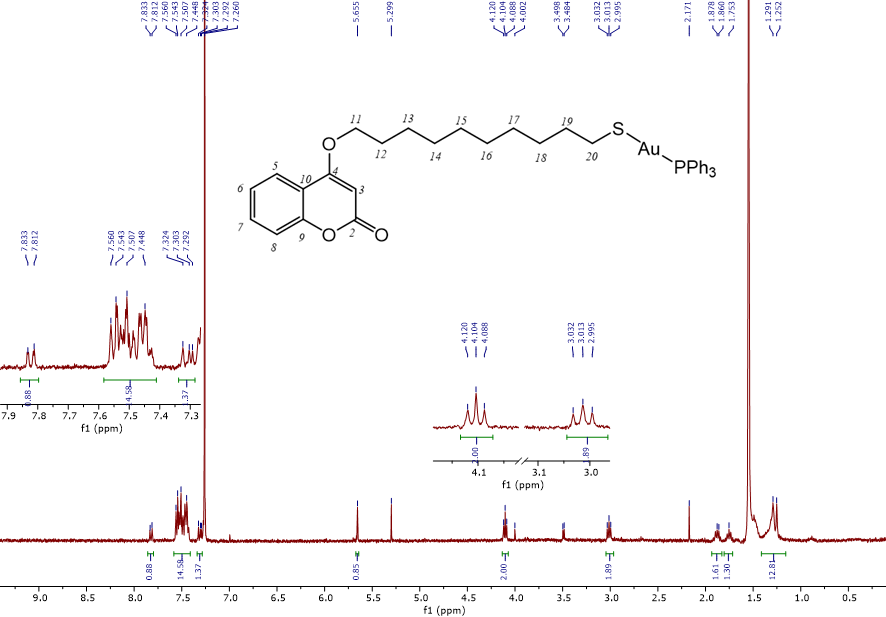


**Figure S24**: ^1^H NMR in CDCl_3_ of AuL2d


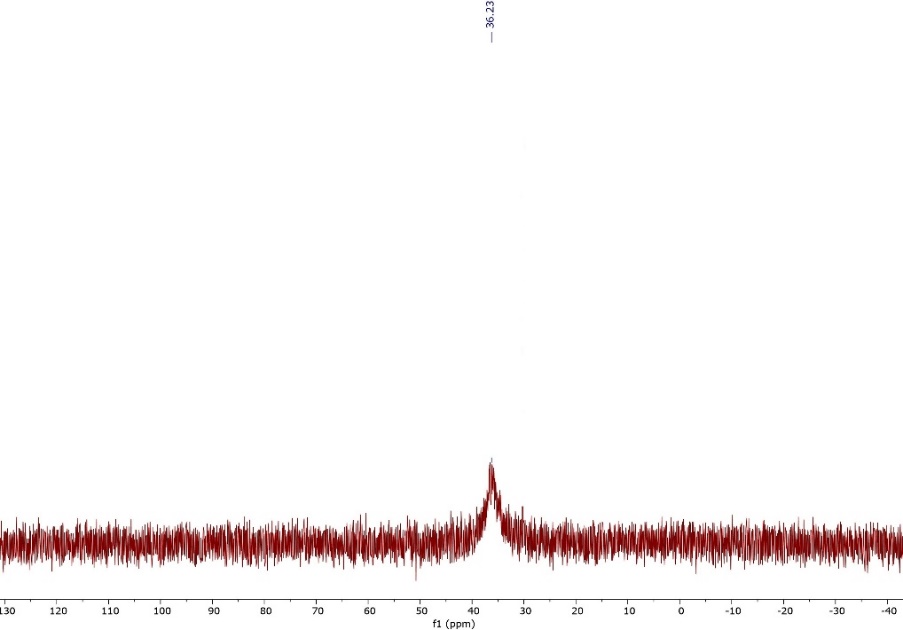


**Figure S25**: ^31^P{^1^H} NMR in CDCl_3_ of AuL2d

**
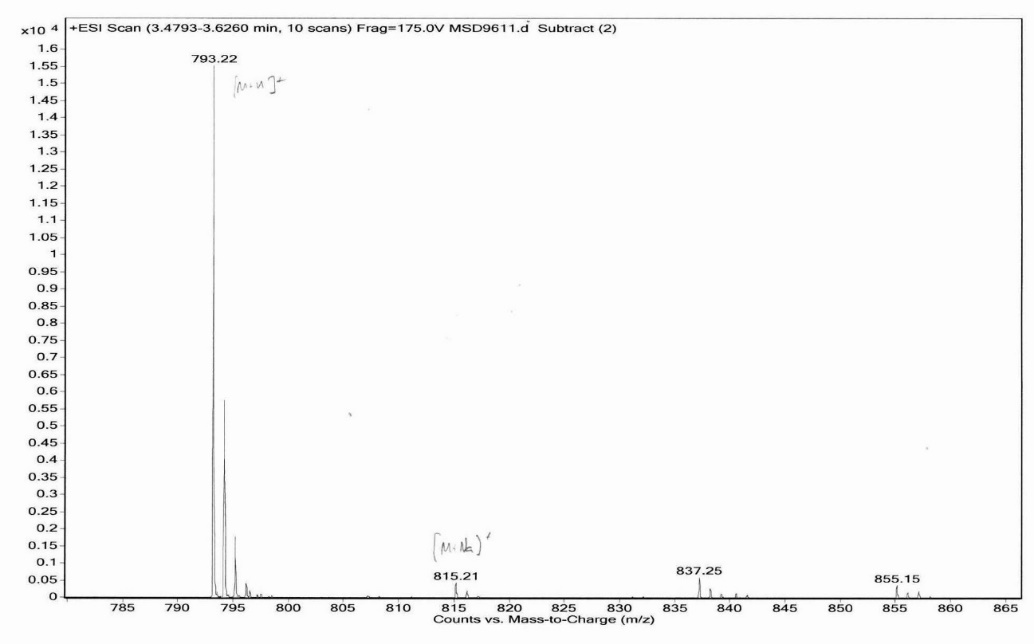
**

**Figure S26**: ESI(+) MS of AuL2d


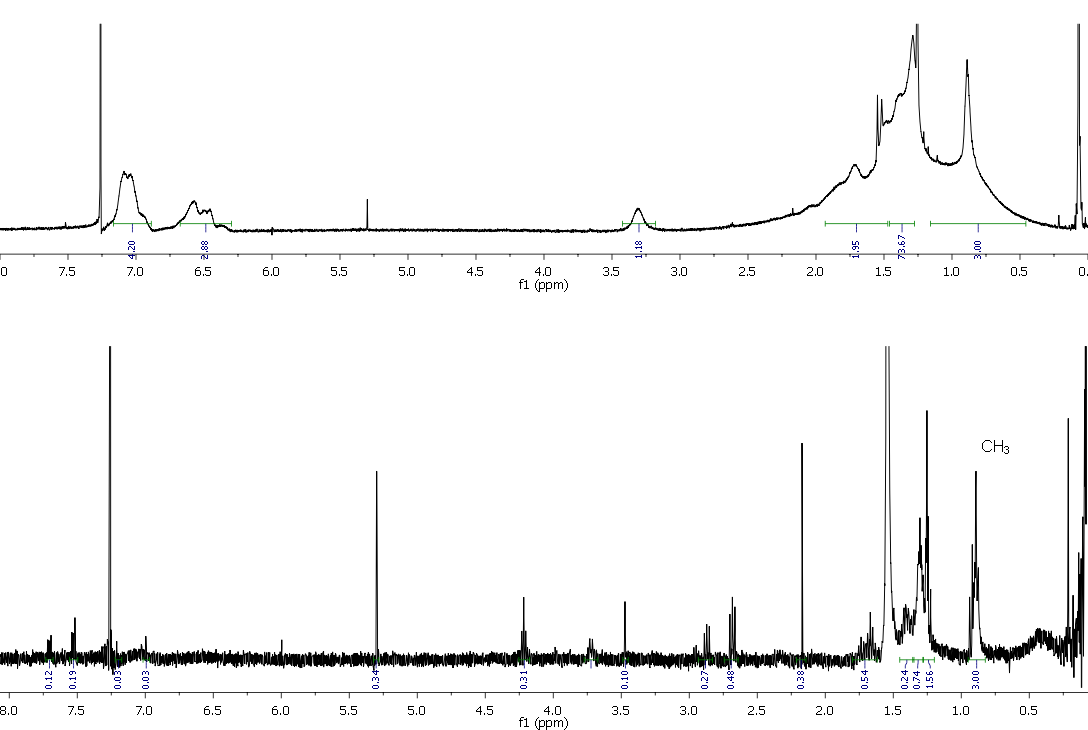


**Figure S27**: ^1^H NMR in CDCl_3_ of AuL1NPs (top) and disulfide ligands after I_2_ decomposition (bottom)

**Figure S28**: ^1^H NMR in CDCl_3_ of Au**L2**NPs (top) and disulfide ligands after I_2_ decomposition (bottom)


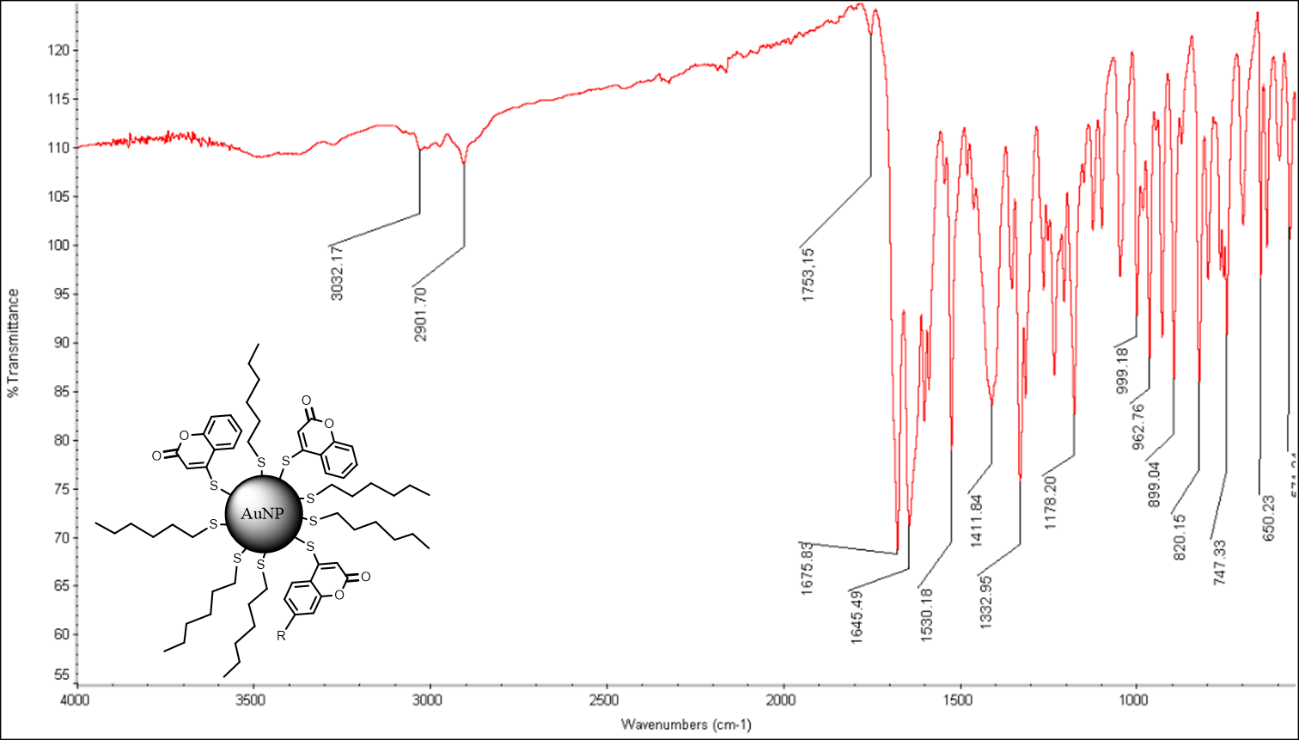


**Figure S29.** IR spectra of AuL1NPs


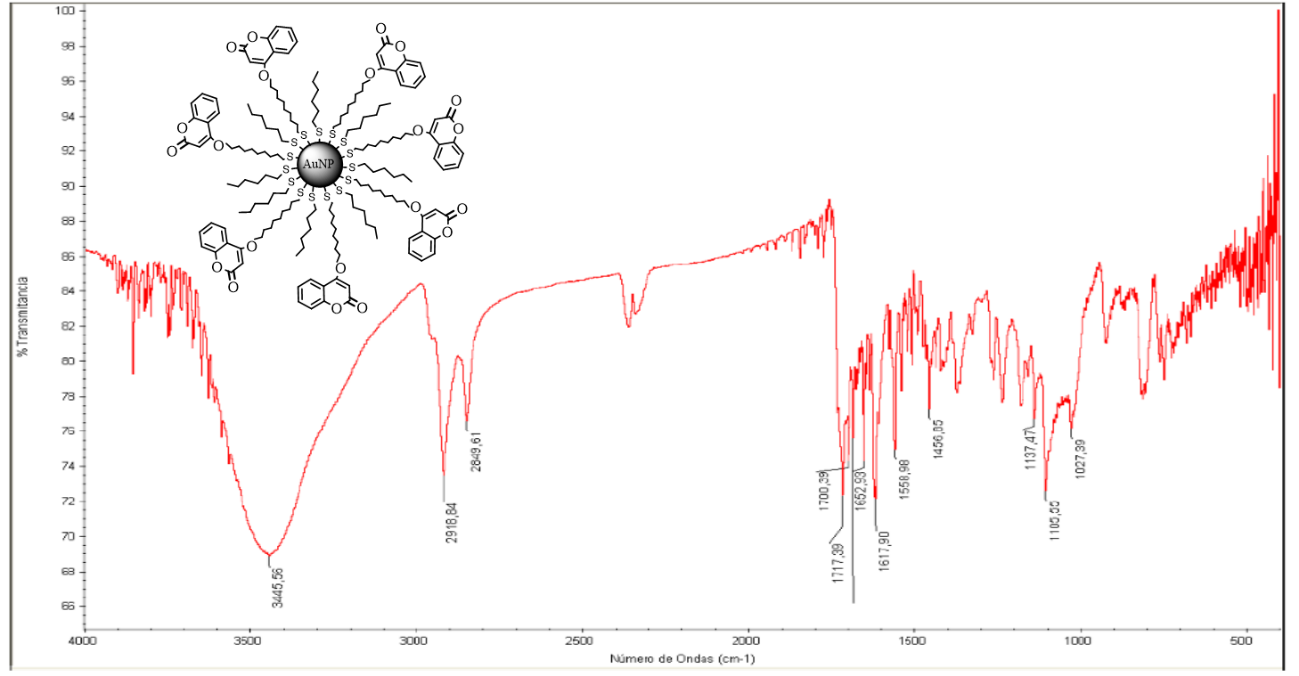


**Figure S30.** IR spectra of AuL2NPs


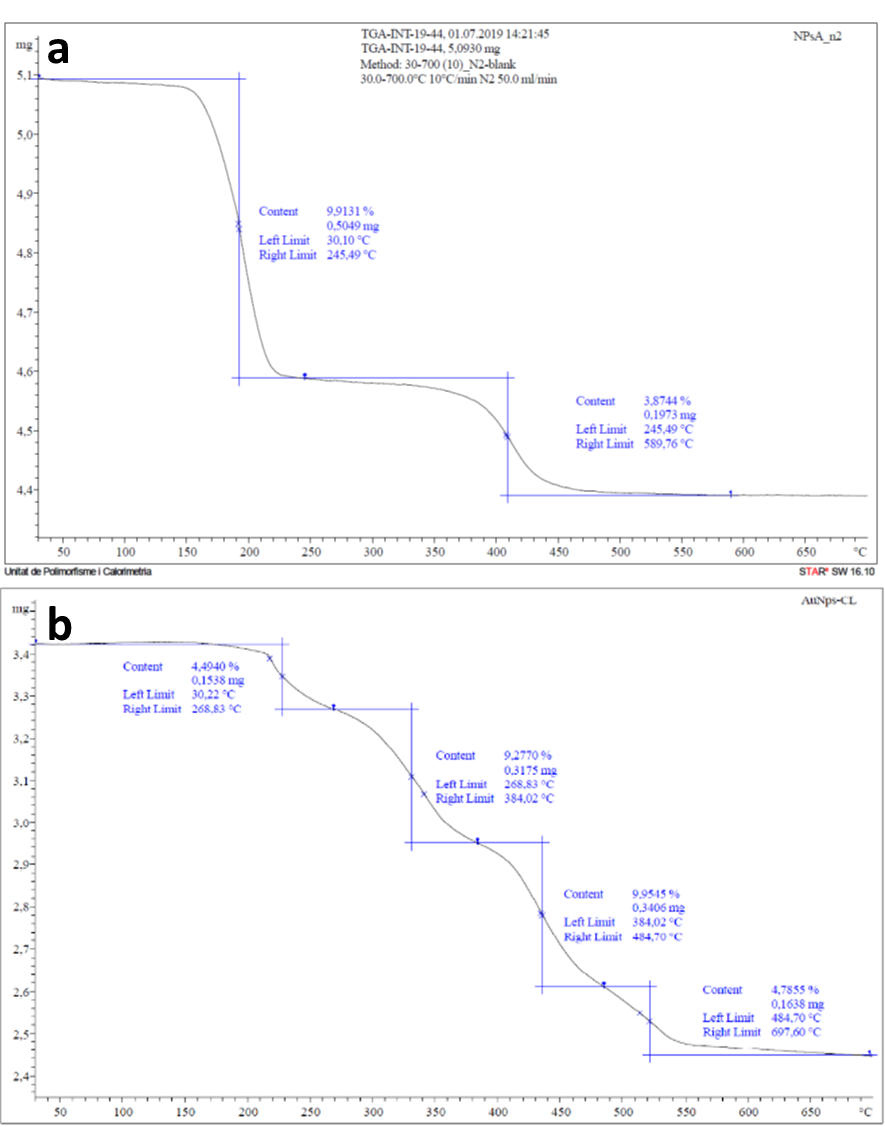


**Figure S31**: TGA of AuL1NPs (a) and AuL2NPs (b)

**
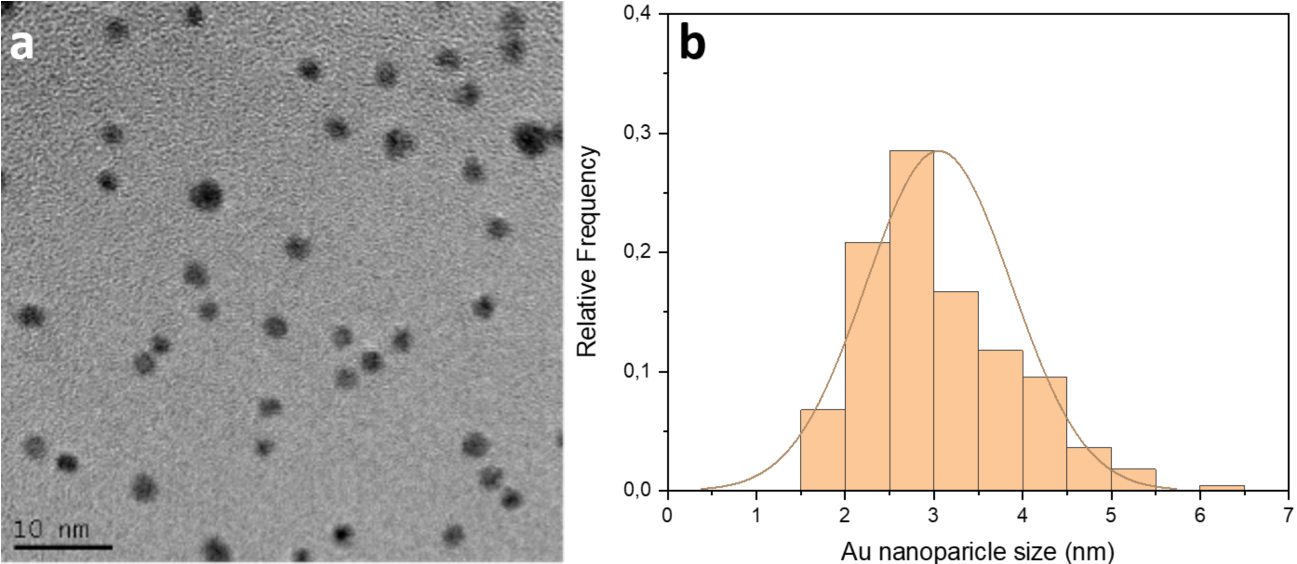
**

**Figure S32.** A representative TEM image of AuL1NPs and AuL2NPs systems. (a) and nanoparticle size distribution. (b)

**
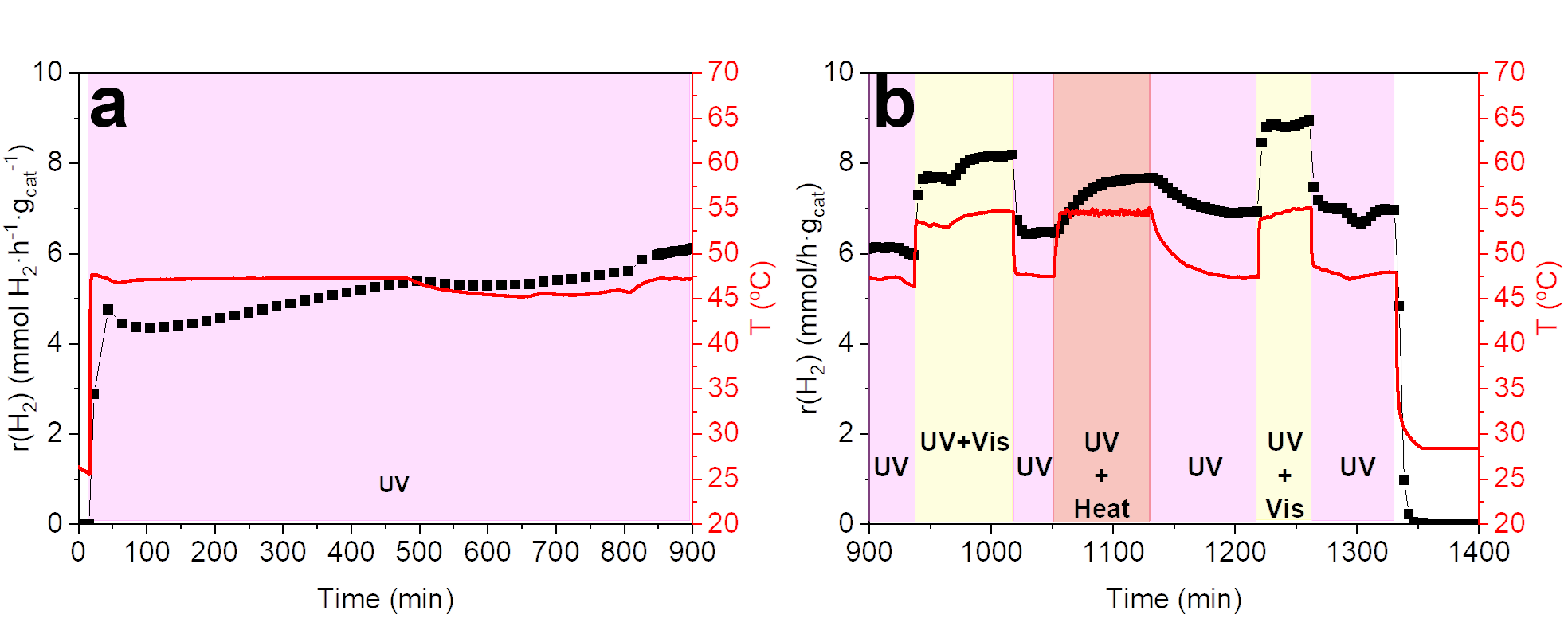
**

**Figure S33.** Hydrogen generation rates. (a) AuL1c/TiO_2_ activation phase. (b) AuL1c/TiO_2_ analysis of light and heat effect.

**
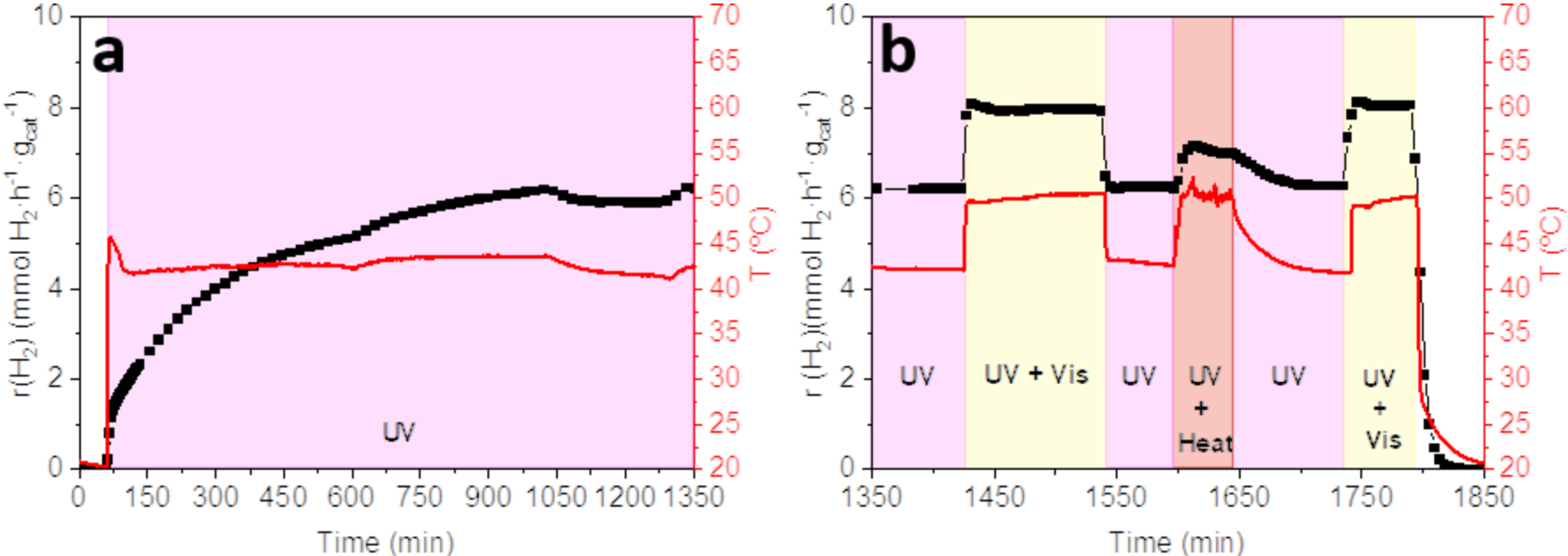
**

**Figure S34.** Hydrogen generation rates. (a) AuL1b/TiO_2_ activation phase. (b) AuL1b/TiO_2_ analysis of light and heat effect.

**
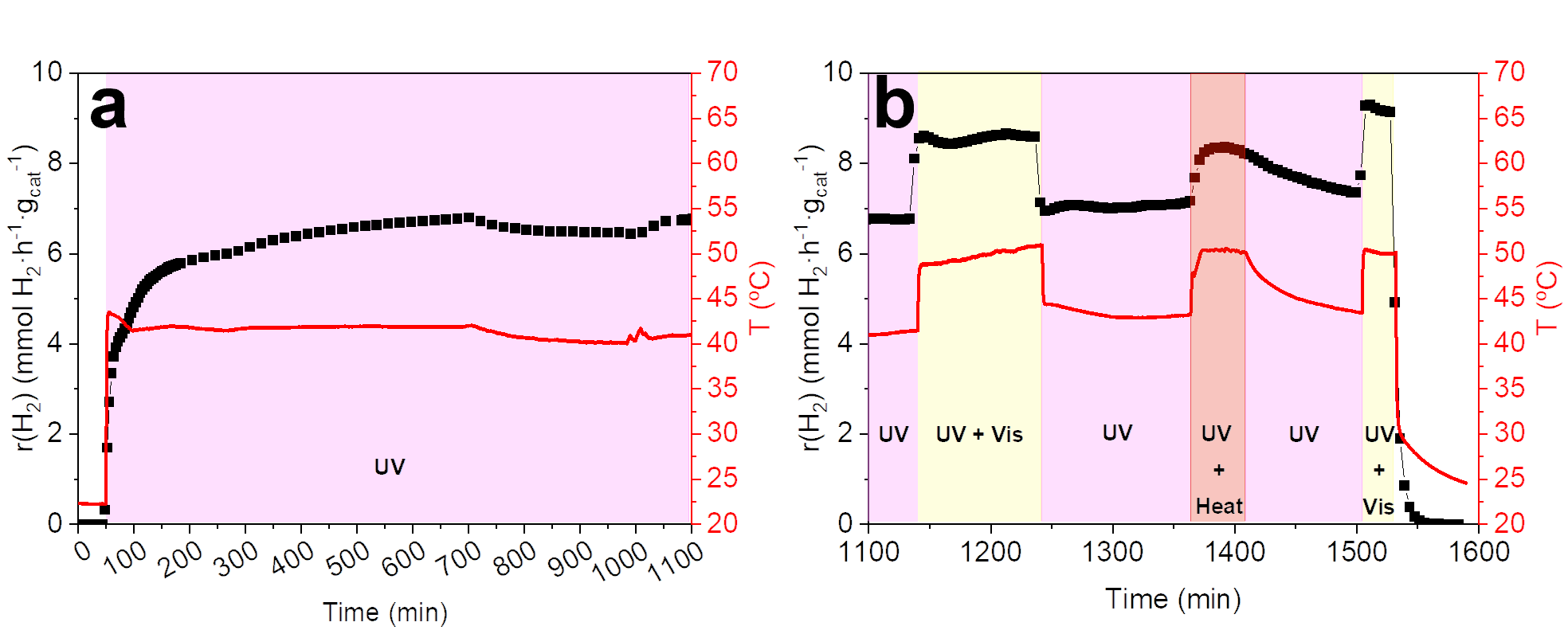
**

**Figure S35**. Hydrogen generation rates. (a) AuL2d/TiO_2_ activation phase. (b) AuL2d/TiO_2_ analysis of light and heat effect.

**
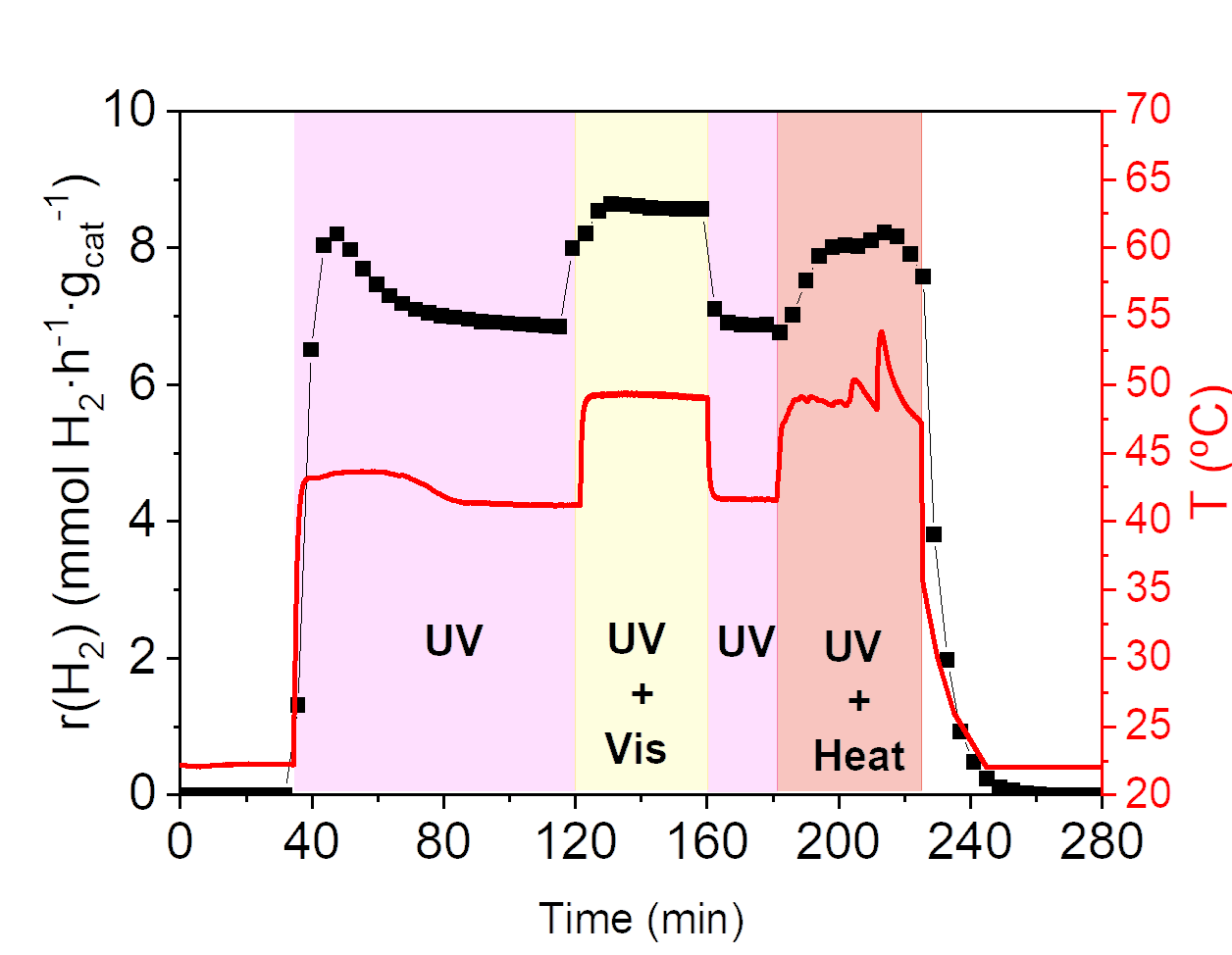
**

**Figure S36.** Hydrogen generation rates of AuL2NPs/TiO_2_ with analysis of light and heat effect.


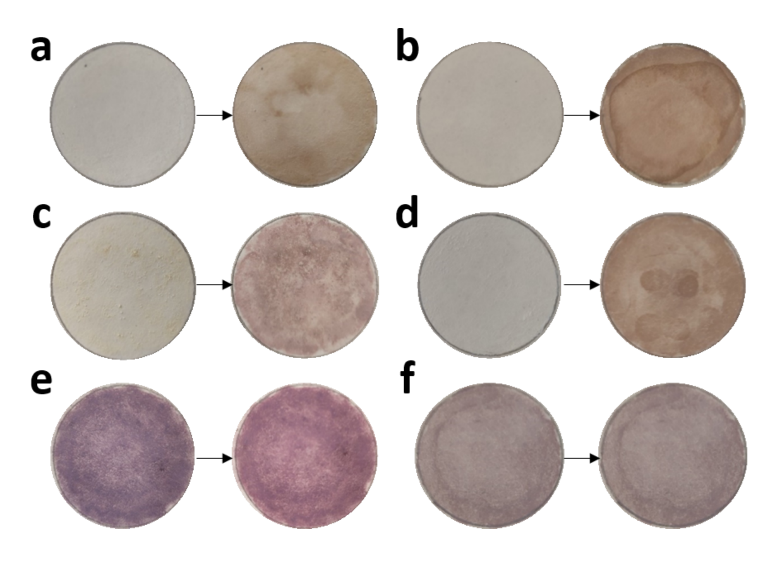


**Figure S37.** Colour of samples before (left) and after (right) reaction. (a) AuL1a/TiO2, (b) AuL1b/TiO2, (c) AuL1c/TiO2, (d) AuL2d/TiO2, (e) AuL1NPs/TiO2, (f) AuL2NPs/TiO2.


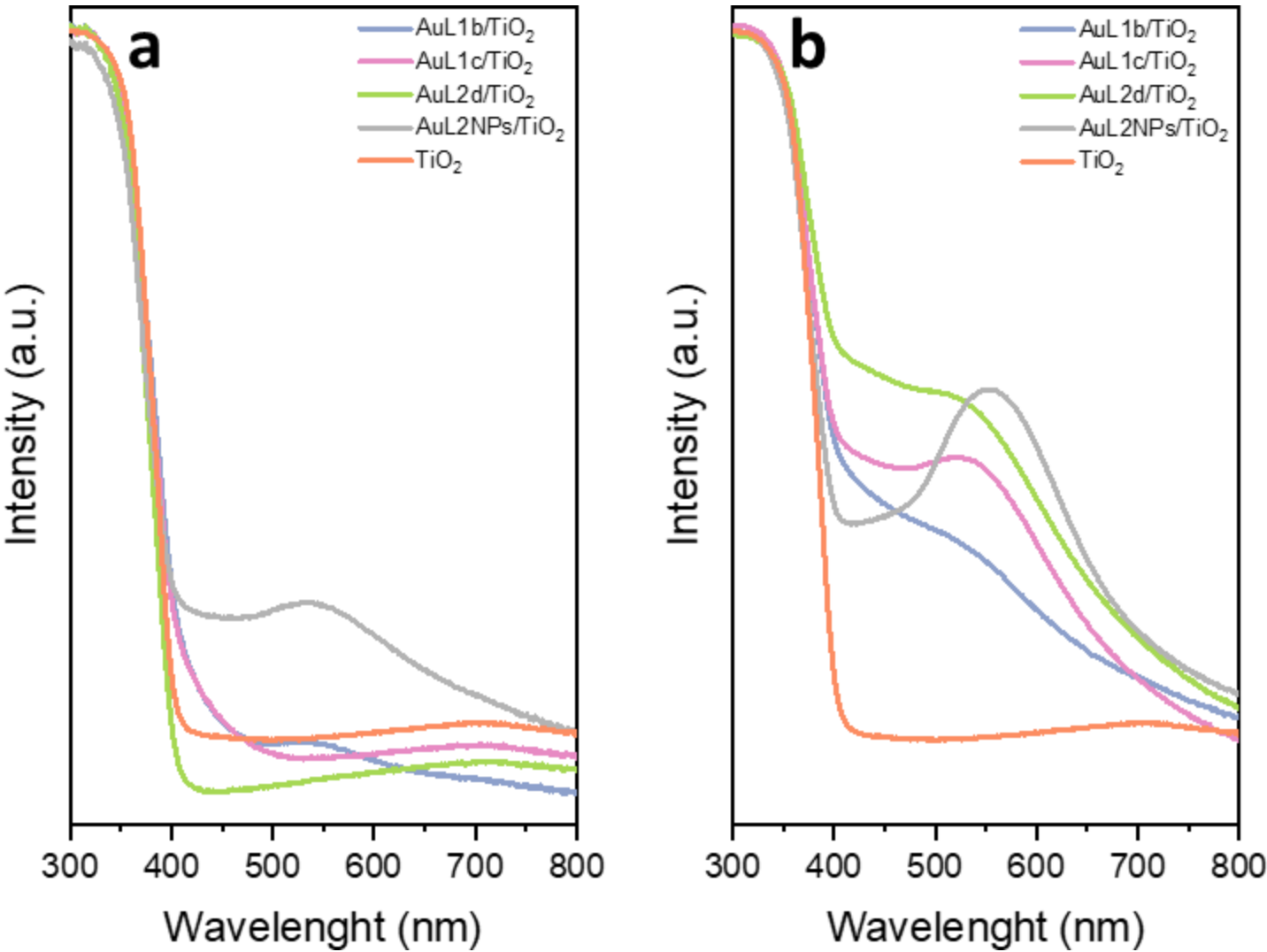


**Figure S38.** UV-Vis spectra for the (a) fresh samples and (b) After reaction samples.

**
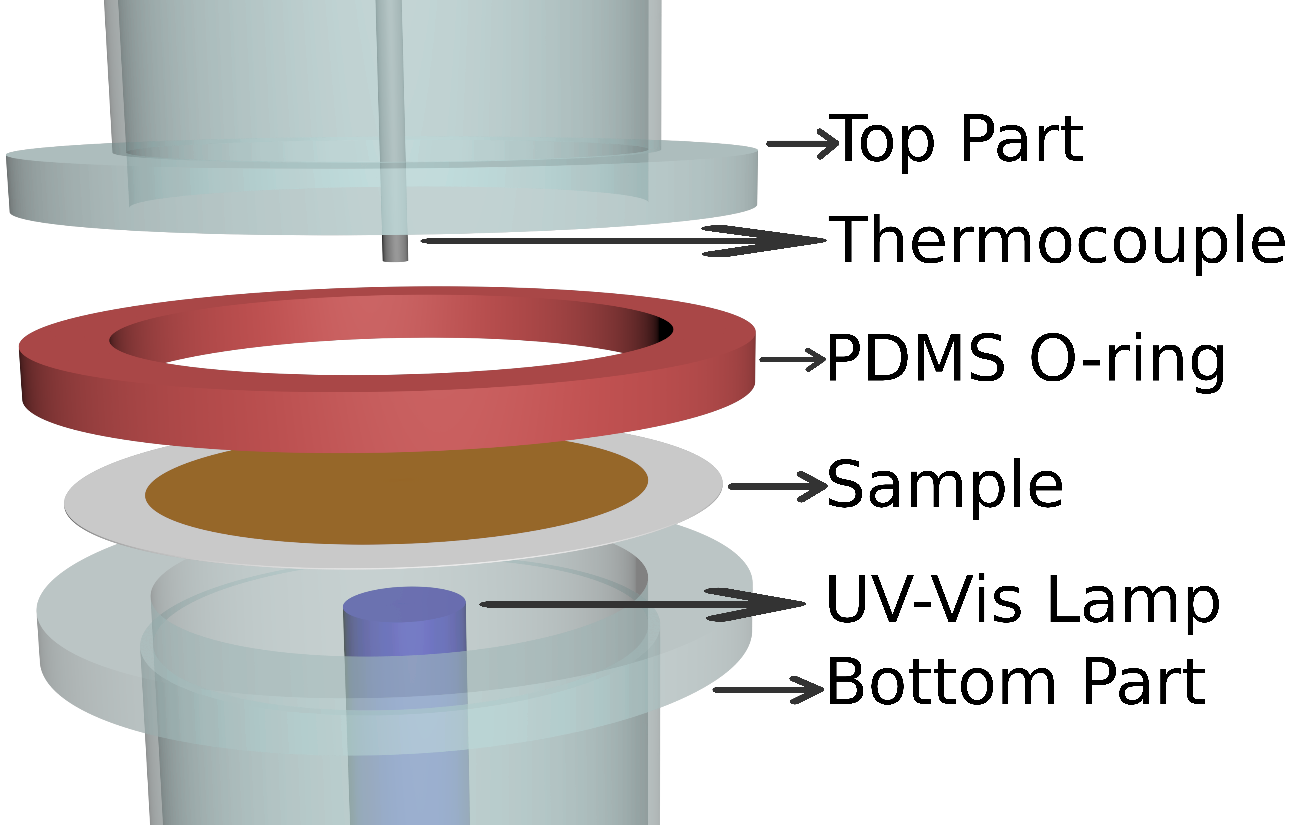
**

**Figure S39.** Schematic view of the photoreactor.

**Supplementary Schemes**

**
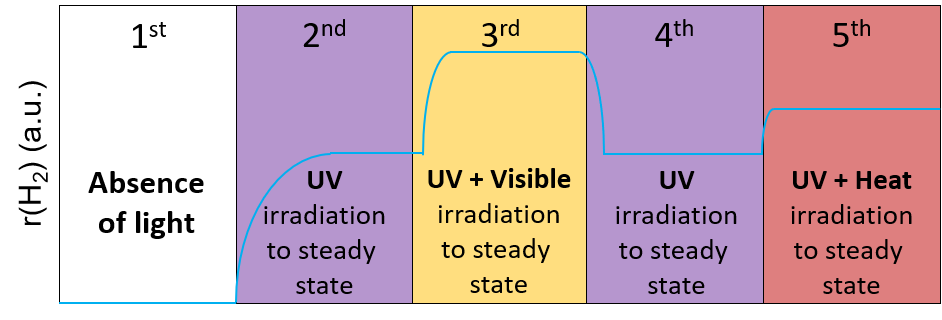
**

**Scheme S1.** Scheme of the photoreaction steps under different light irradiation conditions.

**Scheme S2.** Synthesis of L2.

**Supplementary Table**

**Table S1.** Atomic ratios calculated for XPS Data

|  | AuL1a/TiO_2_  Fresh | AuL1a/TiO_2_  After reaction | AuL1NPs/TiO_2_ Fresh | AuL1NPs/TiO_2_ After reaction |
| --- | --- | --- | --- | --- |
| Au/Ti | 0.016 | 0.010 | 0.006 | 0.007 |
| C/Ti | 1.06 | 1.55 | 0.80 | 1.00 |

**Characterization data of ligands complexes and systems:**

**L1:** ^1^H NMR (CDCl_3_, 400 MHz): δ 7.64 (dd, ^3^J_HH_= 8 Hz, ^4^J_HH_= 1.6 Hz, 1H, H*5*), 7.59 (ddd, ^3^J_HH_= 8.4 Hz, ^3^J_HH_= 7.2 Hz, ^4^J_HH_= 1.6 Hz, 1H, H*7*), 7.36-7.30 (m, 2H, H*8*, *H6*), 6.42 (s, 1H, H*3*), 3.86 (s, 1H, SH). ^13^C{^1^H} NMR (CDCl_3_, 101 MHz): δ 158.8 (^2^C), 152.7 (^9^C), 151.7 (^10^C), 132.9 (^7^CH), 124.8(^5^CH), 124.5 (^6^CH), 118.1 (^4^C), 117.5 (^8^CH), 112.4 (^3^CH).

**L2**: IR ν(cm^-1^): 2923 (C_sp3_-H), 2844 (C_sp3_-H), 2574 (S-H), 1701 (C=O), 1609 (C=C), 1099 (C-O-C). ^1^H NMR (400 MHz, CDCl_3_), δ (ppm): 7.82 (dd, J = 8.0, 1.6 Hz, 1H, H*5*), 7.55 (ddd, J = 8.4, 7.2, 1.6 Hz, 1H, H*7*), 7.32 (d_br_, J= 8.4 Hz, 1H, H*8*) 7.28 (*p*td, J= 7.2, 1.2 Hz, 1H, H*6*), 5.67 (s, 1H, H*3*), 4.13 (t, J = 6.4 Hz, 2H, -OCH_2_), 2.53 (*p*q, J= 7.2 Hz, 2H, -C*H_2_*SH), 1.90 (m, 2H, ^12^C*H*_2_), 1.63-1.31 (m, 15H, ^13-19^C*H*_2_, S*H*).

^13^C{^1^H} NMR (100.6 MHz, CDCl_3_), δ(ppm): 165.7 (s, ^4^C), 163.0 (s, ^2^C), 153.4 (s, ^9^C), 132.3 (s, ^7^*C*H), 123.8 (s, ^6^*C*H), 122.9 (s, ^5^*C*H), 116.8 (s, ^8^*C*H), 115.8 (s, ^10^C), 90.4 (s, ^3^*C*H), 69.4 (s, ^11^*C*H_2_O), 33.9 (s, ^12^*C*H_2_), 29.4 (s, CH_2_), 29.2 (s, CH_2_), 29.0 (s, CH_2_), 28.5 (s, ^12^CH_2_), 28.3 (s, CH_2_), 25.9 (s, CH_2_), 24.6 (s, ^20^*C*H_2_SH).

MS ESI(+) for C_19_H_26_O_3_S, m/z: 335.17 (335.17 calc.) [M + H]^+^, 357.15 (357.15 calc.) [M + Na]^+^.

**AuL1a**: IR ν(cm^-1^): 3026 (C_sp2_ -H), 2907 (C_sp3_ -H), 1678, 1645 (C=O), 1333 (C-N), 1178 (C-O). ^1^H NMR (CDCl_3_, 400 MHz), δ (ppm): 8.14 (dd, J= 8.4, 1.6 Hz, 1H, H*5*), 7.49 (td, J = 8, 1.6 Hz, 1H, H*7*), 7.29 (d, J = 7.6 Hz, 1H, H*8*), 7.27 (m, 1 H, H*6*), 6.86 (s, 1 H, H*3*), 5.84 (d, J = 14 Hz, 1H, NC*^d^H*_2_N), 5.75 (dd, J= 16, 8 Hz, 1 H, NC*^a^H_2_*P), 5.00 (d, J= 14 Hz, 1 H, NC*^e^H_2_*N), 4.76 (dd, J= 15, 11 Hz, 1H, NC*^c^H_2_*P), 4.71 (d, J= 14 Hz, 1 H, NC*^e’^H_2_*N), 4.23 (td, J= 15, 3 Hz, 1H, NC*^c’^H_2_*P), 4.12 (d, J= 14 Hz, 1H, NC*^d’^H_2_*N), 3.96 (s, 2 H, NC*^b^H_2_*P), 3.61 (d_br_, J= 16 Hz, 1 H, NC*^a’^H_2_*P), 2.16 and 2.14 (s + s, 6H, CH_3_). ^13^C{^1^H} NMR (CDCl_3_, 101 MHz), δ (ppm): 131.7 (^7^CH), 127.2 (^5^CH), 123.7 (^6^CH), 116.8 (^8^CH), 113.9 (^3^CH), 67.4 (s, ^e^CH_2_), 62.1 (s, ^d^CH_2_), 49.8 (s, ^b^CH_2_), 44.8 (s, ^c^CH_2_), 39.4 (s, ^a^CH_2_), 21.2 (s, CH_3_), 21.2 (s, CH_3_). ^31^P{^1^H} NMR (CDCl_3_, 162 MHz), δ (ppm): -24.8. MS ESI(+) for C_18_H_21_AuN_3_O_4_PS, m/z: 604.07 [M+H]^+^ (calc.:604.07 g/mol).

**AuL1b**: IR ν(cm^-1^): 3032 (C_sp2_ -H), 2907 (C_sp3_ -H), 1679, 1648 (OC=O), 1329 (C-N). ^1^H NMR (CDCl_3_, 400 MHz), δ (ppm): 8.14 (dd, J= 8 Hz, J=1.6Hz, 1 H, H*5*), 7.49 (td, J= 8 Hz, J=1.6Hz, 1 H, H*7*), 7.29 (d, ^3^J_H,H_= 8 Hz, 1 H, H*8*), 7.27 (m, 1 H, H*6*), 6.86 (s, 1 H, H*3*), 5.80 (d, J= 14 Hz, 2 H, NC*^d^H*_2_N), 5.72 (dd, J = 16 Hz, J= 8 Hz, 2 H, NC*^a^H_2_*P), 4.97 (d, ^2^ J_H,H_= 14 Hz, 2 H, NC*^e^H_2_*N), 4.70 (dd, J= 15, J=11 Hz, 2 H, NC*^c^H_2_*P), 4.67 (d, J= 14 Hz, 2 H, NC*^e’^H_2_*N), 4.25 (dt, J= 15 Hz, J= 3 Hz, 2 H, NC*^c’^H_2_*P), 4.09 (d, J= 14 Hz, 2 H, NC*^d’^H*_2_N), 3.97 (s, 4 H, NC*^b^H_2_*P), 3.67 (d_br_, J= 16 Hz, 2 H, NC*^a’^H_2_*P), 2.07 and 2.06 (s+ s, 12H, CH_3_). ^13^C{^1^H} NMR (CDCl_3_, 101 MHz), δ (ppm): 170.2 (NCO), 169.8 (NCO), 163.3 (CO), 159.2 (^10^C), 152.5 (^9^C), 132.0 (^7^CH), 127.1 (^5^CH), 124.1 (^6^CH), 122.3 (^4^C), 117.0 (^8^CH), 114.0 (^3^CH), 67.4 (d, ^3^J_CP_= 5.1 Hz, ^e^CH_2_), 62.2 (d, ^3^J_CP_= 5.5 Hz, ^d^CH_2_), 49.5 (d, ^1^J_CP_= 28 Hz, ^b^CH_2_), 44.9 (d, ^1^J_CP_= 29 Hz, ^c^CH_2_), 39.6 (d, ^1^J_CP_= 30 Hz, ^a^CH_2_), 21.9 and 21.5 (s+ s, CH_3_). ^31^P{^1^H} NMR (CDCl_3_, 162 MHz), δ (ppm): -28.4. MS ESI(+) for C_27_H_37_Au_2_ClN_6_O_6_P_2_S, m/z: 1029.13 (calc. 1029.13) [M-Cl]^+^, 604.07 [M-AuCl(DAPTA)+H]^+^.

**AuL1c**: IR ν(cm^-1^): 3053 (C_sp2_-H), 1699 (C=O), 1623, 1601 (C=C), 1479, 1435 (CC_Ar_). ^1^H NMR (CDCl_3_, 400 MHz), δ (ppm): 8.26 (dd, J= 8.4, 1.6 Hz, 1H, H_5_), 7.58-7.45 (m, 16H, H_7_ + Ph), 7.28-7.24 (m, 2H, H_8_, H_6_), 7.01 (s, 1 H, H_3_). ^31^P{^1^H} NMR (CDCl_3_, 162 MHz): δ 38.3 (s_br_). MS ESI(+) for C_27_H_20_AuO_2_PS, m/z: 637.0657 (637.0665 calc.) [M+H]^+^, 659.0466 (659.0479 calc.) [M+Na]^+^, 721.1483 (721.1483 calc.) [Au(PPh_3_)_2_]^+^.

**AuL1d**: IR ν(cm^-1^): 3081 (C_sp2_-H), 2921 (C_sp3_-H), 2847 (C_sp3_-H), 1723 (C=O), 1622 (C=C). ^1^H NMR (CDCl_3_, 400 MHz), δ (ppm): 7.82 (d, J = 8.0 Hz, 1H, H*5*), 7.54-7.45 (m,17H, H*7*, PPh_3_), 7.32 (m, 2H, H*6*, H*8*), 5.65 (s, 1H, H*3*), 4.10 (t, J = 6.4 Hz, 2H, -OCH_2_), 3.01 (t, J = 8.0 Hz, 2H, C*H_2_*SH), 1.88 (m, 2H, ^12^C*H*_2_), 1.75-1.21 (m, 14H, ^13-19^C*H*_2_). ^31^P{^1^H} NMR (CDCl_3_, 400 MHz): δ (ppm) 36.2 (s_br_). MS ESI(+) for C_37_H_40_AuO_3_PS, m/z: 793.22 (793.22 calc.) [M+H]^+^, 815.21 (815.20 calc.) [M+Na]^+^, 721.15 (721.15 calc.) [Au(PPh_3_)_2_]^+^, 1251.27 (1251.27 calc.) [M+(AuPPh_3_)]^+^.

**AuL1NPs:** IR (KBr): 3032 (C_sp2_-H), 2901 (C_sp3_ -H), 1675, 1645 (C=O), 1411 (CC). ^1^H RMN (CDCl_3_, 400 MHz), δ (ppm): broad signals at 7.08 (CH), 6.58 (CH), 2.04-0.89 (m_br_, CH_2_, CH_3_). ^1^H NMR of organics after NPs decomposition with I_2_ (CDCl_3_, 400 MHz), δ (ppm): 7.64 (^7^CH), 7.63 (^5^CH), 7.46 (^6^CH), 7.45 (^8^CH), 6.93 (^3^CH), 2.80 (t, CH_2_S_2_R), 2.61 (t, CH_2_S_2_R’), 0.82 (t, CH_3_). TGA: 86% Au

**AuL2NPs**: IR ν(cm^-1^): 2919 (C_sp2_-H), 2849 (C_sp2_-H), 1717 (C=O), 1618 (C=C). ^1^H RMN (CDCl_3_, 400 MHz), δ (ppm): broad signals at 7.75 (H*5*), 7.52 (H*7*), 7.14 (H*6*, H*8*), 5.60 (H*3*), 4.06 (CH_2_O), 2.68 (very small triplet, CH_2_S), 2.05-0.85 (m_br_). ^1^H RMN of organics after I_2_ decomposition (CDCl_3_, 400 MHz), δ (ppm): 7.82 (d, J=8 Hz, 0.8H, H*5*), 7.55 (t, J=8 Hz, 0.8 H, H*7*), 7.32 (d, J= 8.4 Hz, H*8*), 7.28 (t, J=7.6 Hz, H*6*), 5.67 (s, H3), 4.13 (t, J = 6.4 Hz, 1.7H, CH_2_O), 2.68 (t, J= 7.2 Hz, 4H, CH_2_S), 1.90 (m, 1.6H, ^12^C*H*_2_), 1.70-1.25 (m, C*H*_2_), 0.89 (t, J=7 Hz, 3 H, CH_3_). Molar ratio 1-hexanothio/L2 1:0.8. TGA: 72%Au.
